# IGF1R acts as a cancer-promoting factor in the tumor microenvironment facilitating lung metastasis implantation and progression

**DOI:** 10.1038/s41388-022-02376-w

**Published:** 2022-06-10

**Authors:** Elvira Alfaro-Arnedo, Icíar P. López, Sergio Piñeiro-Hermida, Marta Canalejo, Carolina Gotera, Jesús Javier Sola, Alejandra Roncero, Germán Peces-Barba, Carlos Ruíz-Martínez, José G. Pichel

**Affiliations:** 1 Lung Cancer and Respiratory Diseases Unit, Center for Biomedical Research of La Rioja (CIBIR), Fundación Rioja Salud, Logroño, Spain; 2 Telomeres and Telomerase Group, Molecular Oncology Program, Spanish National Cancer Centre (CNIO), Madrid, Spain; 3 IIS Fundación Jiménez Díaz, Madrid, Spain; 4 Spanish Biomedical Research Networking Centre, CIBERES, Madrid, Spain; 5 Pathological Anatomy Service, Hospital Universitario San Pedro, Rioja Salud, Logroño, Spain; 6 Pneumology Service, Hospital Universitario San Pedro, Rioja Salud, Logroño, Spain

**Keywords:** Cancer microenvironment, Predictive markers

## Abstract

Given the long-term ineffectiveness of current therapies and late-stage diagnoses, lung cancer is a leading cause of malignant diseases. Tumor progression is influenced by cancer cell interactions with the tumor microenvironment (TME). Insulin-like growth factor 1 receptor (IGF1R) was reported to affect the TME; however, the role of IGF1R in lung TME has not been investigated. First, we assessed IGF1R genomic alterations and expression in NSCLC patient tissue samples, as well as IGF1R serum levels. Next, we performed tumor heterotopic transplantation and pulmonary metastases in IGF1R-deficient mice using melanoma and Lewis lung carcinoma (LLC) cells. Herein we report increased amplification and mRNA expression, as well as increased protein expression (IGF1R/p-IGF1R) and IGF1R levels in tumor samples and serum from NSCLC patients, respectively. Moreover, IGF1R deficiency in mice reduced tumor growth, proliferation, inflammation and vascularization, and increased apoptosis after tumor heterotopic transplantation. Following induction of lung metastasis, IGF1R-deficient lungs also demonstrated a reduced tumor burden, and decreased expression of tumor progression markers, p-IGF1R and p-ERK1/2. Additionally, IGF1R-deficient lungs showed increased apoptosis and diminished proliferation, vascularization, EMT and fibrosis, along with attenuated inflammation and immunosuppression. Accordingly, IGF1R deficiency decreased expression of p-IGF1R in blood vessels, fibroblasts, tumor-associated macrophages and FOXP3^+^ tumor-infiltrating lymphocytes. Our results demonstrate that IGF1R promotes metastatic tumor initiation and progression in lung TME. Furthermore, our research indicates that IGF1R could be a potential biomarker for early prediction of drug response and clinical evolution in NSCLC patients.

## Introduction

Lung cancer is a leading cause of malignant diseases and the most common cause of cancer-related death worldwide with a 5-year survival rate of about 21% [1, 2]. The predominant lung cancer subtype, non-small-cell lung cancer (NSCLC), accounts for 80% of lung cancer-associated deaths, and about 70% of patients have locally advanced or metastatic disease at the time of diagnosis. Moreover, the lung is one of the most common sites for cancer metastases. Distant tumor metastasis to the lung changes staging, clinical prognosis and treatment options of the original tumor and severely decreases survival rate [3–5].

Tumor masses consist of a heterogeneous population of cancer cells and a variety of resident and infiltrating host cells, secreted factors, and extracellular matrix proteins, collectively known as the tumor microenvironment (TME). Among others, tissue-resident and peripherally recruited immune cells, fibroblasts, and endothelial cells are key elements within the TME. Tumor progression is profoundly influenced by cancer cells interactions with the TME, which ultimately determine whether the primary tumor is eradicated, metastasizes, or establishes dormant micrometastases. Moreover, tumor metastasis requires the development of a pre-metastatic niche suitable for a subpopulation of tumor cells to colonize and develop into metastases with their own TME. Since the TME can also shape therapeutic responses and resistance, a deeper understanding of TME characteristics and function is required for developing new strategies for targeting its components in patients with primary and metastatic tumors [6–8].

The insulin-like growth factor 1 receptor (IGF1R) is a ubiquitously expressed membrane-bound tyrosine kinase receptor that recognizes its two major ligands IGF1 and IGF2, and controls multiple essential cellular functions [9]. IGF activity is highly relevant in several chronic lung pathologies, including lung cancer [10–12]. IGF1R signaling has been profusely implicated as a critical contributor to cancer cell proliferation, survival, migration, and resistance to anticancer therapies, thus targeting IGF signaling is an attractive therapeutic strategy. In this regard, IGF1R is currently being evaluated as a pharmacological target in clinical trials for oncologic patients, including NSCLC [13, 14]. However, the role IGF1R plays in implantation and progression of lung metastases in the TME has not been evaluated in the context of NSCLC. The Lewis lung carcinoma (LLC) model is the only reproducible syngeneic model for NSCLC [15]. Here, we studied the implication of IGF1R as a cancer-promoting factor in the TME by analyzing tumor samples from NSCLC patients, and generating LLC models by performing heterotopic transplantation or pulmonary metastasis in the context of IGF1R deficiency.

## Results

### Increased *IGF1R* amplification and mRNA expression, as well as upregulation of *IGF1R* protein expression in tumor samples and serum in NSCLC patients

**Fig. 1:**
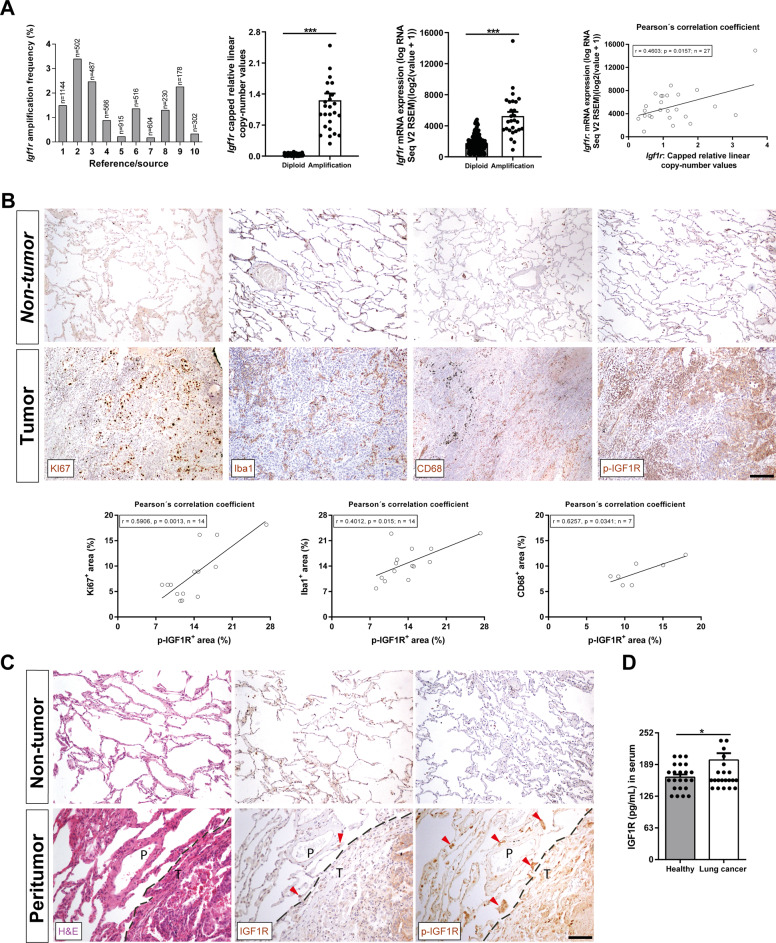
Increased *IGF1R* amplification and mRNA expression, as well as upregulation of IGF1R protein expression in NSCLC patient tumor samples and serum. **A** *IGF1R* gene amplification frequency, copy number values and mRNA expression levels, as well as Pearson´s correlation of *IGF1R* copy number values with mRNA expression in tumor samples from NSCLC patients. Data were obtained from the cBio Cancer Genomics Portal (cBioPortal). **B** Representative immunostains for Ki67 (proliferation), Iba1 (macrophages), CD68 (tumor-associated macrophages, TAMs) and p-IGF1R in tumoral and non-tumoral tissues from NSCLC patients (*n* = 7–14; Scale bar: 125 µm), and respective Pearson´s correlations of p-IGF1R^+^ area with Ki67^+^,Iba1^+^ and CD68^+^ areas (%). **C** Representative stains for H&E and immunostains for IGF1R and p-IGF1R in peritumoral lung tissues from NSCLC patients (dashed lines indicate limits between peritumoral (P) and tumoral (T) areas. Note accumulations of infiltrated immune cells in P (red arrows). **D** Serum IGF1R levels from NSCLC patients and controls (*n* = 24). In bar graphs, data are expressed as mean ± SEM. *p < 0.05; ***p < 0.001 (Mann–Whitney U test for comparing two groups and Pearson´s correlation coefficient).

To explore genomic alterations and mRNA expression of *IGF1R* in patients with NSCLC, we used the cBio Cancer Genomics Portal (cBioPortal). Overall, data obtained from different studies included in the cBioPortal cancer database, showed an increased *IGF1R* amplification frequency with an average of 1.385%. In addition, copy number values and mRNA expression were significantly increased in tissue samples from NSCLC patients in which IGF1R was found amplified with respect to diploid tissue. We also observed that the increase of IGF1R mRNA expression correlated with copy number values (Pearson´s correlation coefficient, *r* = 0.4603, *p* = 0.0157) (Fig. 1A and Supplementary Table 1). To complement this data IGF1R was assessed in tissue samples and serum from our own NSCLC patient cohort. We show that the increase of p-IGF1R expression in tumoral lung tissues correlated with proliferation (Ki67), macrophage (Iba1) and tumor-associated macrophage, TAMs (CD68) presence (Pearson´s correlation coefficient, *r* = 0.5906, *p* = 0.0013; *r* = 0.4012, *p* = 0.015; and *r* = 0.6257, *p* = 0.0341, respectively) (Fig. 1B). We also report overexpression of both IGF1R and p-IGF1R, although this increase was more evident in the case of p-IGF1R in peritumoral lung tissue from NSCLC patients, corresponding to accumulations of infiltrated immune cells (Fig. 1C). Finally, serum IGF1R protein levels evaluated by ELISA revealed a significant increase in NSCLC patients compared to healthy controls (Fig. 1D).

### IGF1R deficiency reduces tumor growth, proliferation, inflammation and vascularization, and increases apoptosis after tumor heterotopic transplantation

**Fig. 2:**
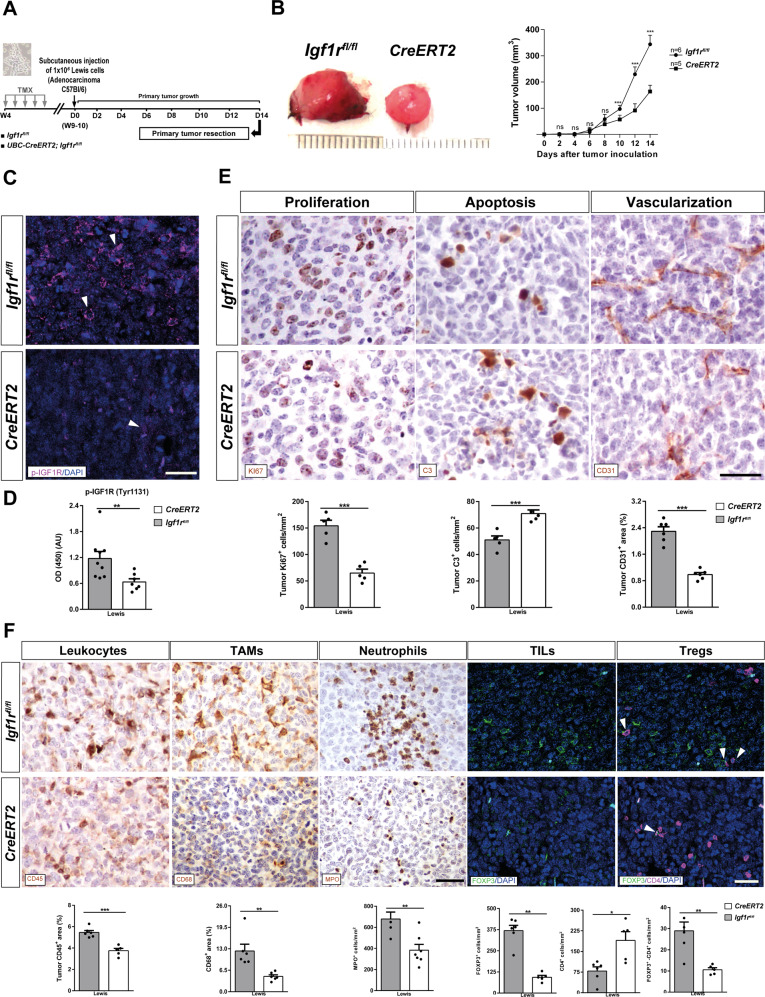
IGF1R deficiency reduces tumor growth, proliferation, vascularization and inflammation, and increases apoptosis after tumor heterotopic transplantation. **A** *UBC-CreERT2;Igf1r*^*fl/fl*^ and *Igf1r*^*fl/fl*^ (controls) female mice were treated with tamoxifen (TMX) for five consecutive days at four weeks (W) of age to induce *Igf1r* gene deletion. Then, mice were subcutaneously injected in their right flanks with 1 × 10^6^ Lewis lung carcinoma (LLC) cells in PBS or an equal volume of PBS. A follow-up of LLC engraftments was performed for 14 days by measuring tumor size on alternate days; primary tumors were resected on day (D) 14. **B** Macroscopic pictures of subcutaneous resected heterotopic tumors (left) and measurements of tumor volumes on alternate days after inoculation of LLC cells (right) in IGF1R-deficient (*CreERT2*) vs. *Igf1*^*fl/fl*^ (control) mice. Representative immunostains for p-IGF1R (magenta; white arrowheads indicate p-IGF1R^+^ cells) (**C**) and quantification of p-IGF1R protein levels (**D**) in sections and homogenates of heterotopic tumors from *CreERT2* vs. *Igf1*^*fl/f*^ mice (*n* = 7–8 mice per group; Scale bar: 30 µm). **E** Representative immunostains for Ki67 (proliferation), C3 (apoptosis), and CD31 (vascularization) (brown) in the tumor microenvironment (TME), and respective quantifications of Ki67^+^ and C3^+^ cells per unit area (mm^2^), and CD31^+^ area (%)in tumor sections from *CreERT2* vs. *Igf1*^*fl/f*^ mice (*n* = 5–6 mice per group; Scale bar: 35 µm). **F** Representative immunostains for CD45 (total leukocytes), CD68 (TAMs) and MPO (neutrophils) (brown), and for FOXP3 (tumor-infiltrating lymphocytes, TILs) (green) and FOXP3-CD4 (T regulatory cells, Tregs) (green-magenta; white arrowheads indicate double FOXP3^+^CD4^+^ cells), as well as respective quantifications of MPO^+^, FOXP3^+^, CD4^+^ and FOXP3^+^CD4^+^ cells per unit area (mm^2^), and CD68^+^ area (%) (brown) in tumor sections from *CreERT2* vs. *Igf1*^*fl/f*^ mice (n = 5–7 mice per group; Scale bars: 30 µm). Quantifications were performed randomly in five different fields. Data are expressed as mean ± SEM. *p < 0.05; **p < 0.01; ***p < 0.001 (Mann–Whitney U test or Student´s *t*-test).

Since IGF1R has been implicated in the pathogenesis of lung cancer by facilitating metastasis, tumor-associated inflammation and immune checkpoint regulation [12, 16], we decided to assess whether IGF1R deficiency in the TME delays implantation and progression in a heterotopic LLC mouse model. For that purpose, *UBC-CreERT2;Igf1r*^*fl/fl*^ and *Igf1r*^*fl/fl*^ female mice were treated with TMX to induce *Igf1r* gene deletion. Then, mice were subcutaneously injected with LLC cells, and follow-up of LLC engraftments was performed for 14 days to assess the tumor volume as detailed in Fig. 2A. LLC cell tumorigenic capacity was evaluated by measuring the heterotopic tumor volume, which was lower in IGF1R deficient (*CreERT2)* vs. *Igf1r*^*fl/fl*^ mice (Fig. 2B). Next, we assessed p-IGF1R by immunofluoresce and ELISA in sections and homogenates of heterotopic tumors. Notably, the apparent reduction of p-IGF1R in heterotopic tumors from IGF1R deficient mice was reflected in reduced p-IGF1R protein levels measured by ELISA (Fig. 2C, D). On the other hand, delayed tumor growth observed in *CreERT2* mice is supported by reduced proliferation (Ki67^+^ cells) and vascularization (CD31^+^ area), as well as increased apoptosis (C3^+^ cells) (Fig. 2E). In addition, heterotopic tumors from *CreERT2* mice showed diminished inflammation demonstrated by reduced presence of total leukocytes (CD45^+^ area), TAMs (CD68^+^ area), neutrophils (MPO^+^ cells), tumor-infiltrating lymphocytes, TILs (FOXP3^+^), as well as T regulatory cells, Tregs (FOXP3^+^CD4^+^ cells). Conversely, CD4^+^ TILs were found increased in heterotopic tumors from *CreERT2* mice (Fig. 2F). Overall, these results indicate that IGF1R deficiency has an antitumoral effect on the lung TME.

### IGF1R deficiency depletes peripheral monocytes, bone marrow neutrophils and leukocyte counts in BALF, and attenuates the increase of serum IL6 and TNFα levels after experimental pulmonary metastasis

**Fig. 3:**
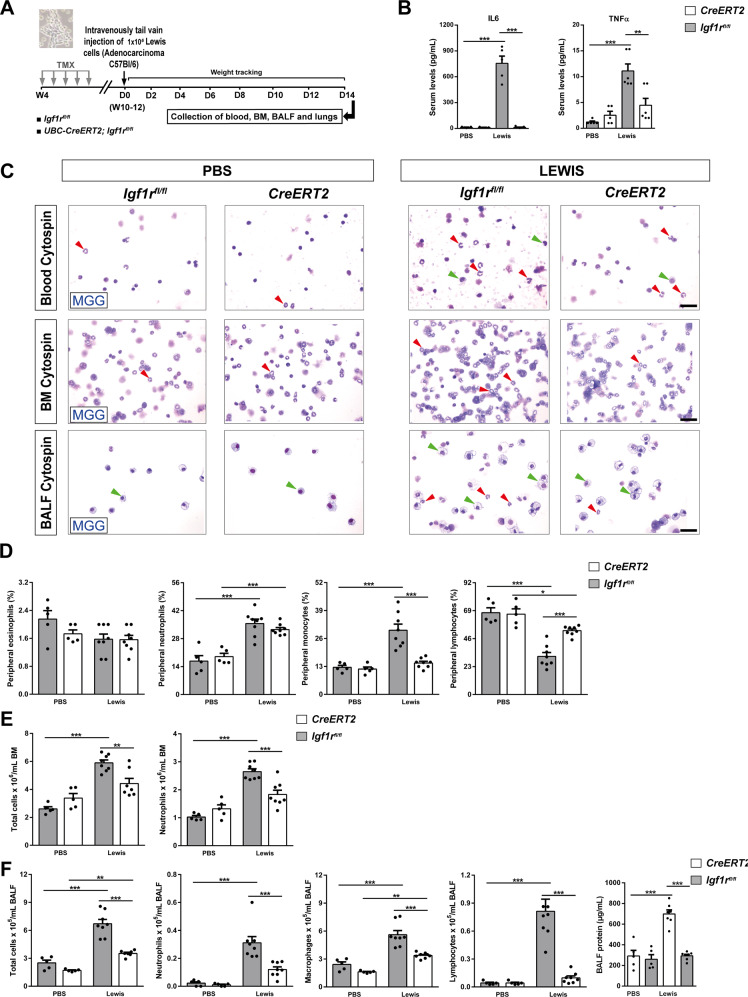
IGF1R deficiency depletes peripheral monocytes, bone marrow neutrophils and leukocyte counts in BALF, and attenuates the increase of serum IL6 and TNFα levels after experimental pulmonary metastasis. **A** *UBC-CreERT2;Igf1r*^*fl/fl*^ and *Igf1r*^*fl/fl*^ female mice were treated with tamoxifen (TMX) for five consecutive days at four weeks (W) of age to induce *Igf1r* gene deletion (*CreERT2*). Then, mice were injected through the lateral tail vein with 1 × 10^6^ Lewis Lung Carcinoma (LLC) cells in PBS or an equal volume of PBS. Collection of blood, bone marrow (BM), BALF and lungs were performed on day (D) 14. **B** Total serum IL6 and TNFα levels from PBS- or LLC-challenged *CreERT2* vs. *Igf1r*^*fl/f*^ mice (*n* = 5–6 mice per group). **C** Representative images showing May-Grünwald Giemsa (MGG) stained peripheral blood, BM and cytospin preparations (red and green arrowheads indicate neutrophils and macrophages, respectively) (Scale bars: 20 µm). Differential cell counts for eosinophils, neutrophils, monocytes and lymphocytes in peripheral blood (**D**), total cells and neutrophils in BM (**E**), and total cells, neutrophils, macrophages and lymphocytes, as well as total protein content in BALF (**F**) from PBS- or LLC-challenged *CreERT2* vs. *Igf1r*^*fl/fl*^ mice (*n* = 5–8 mice per group). Data are expressed as mean ± SEM. *p < 0.05; **p < 0.01; ***p < 0.001 (Dunn–Sidak test for multiple comparisons).

To determine the effect of IGF1R deficiency on key components of the lung TME, we performed an experimental pulmonary metastasis model. *UBC-CreERT2;Igf1r*^*fl/fl*^ and *Igf1r*^*fl/fl*^ female mice were treated with TMX to induce *Igf1r* gene deletion (*CreERT2* mice). Then, mice were intravenously injected with LLC cells in PBS or equal volume of PBS (Fig. 3A). IL6 and TNFα serum levels demonstrated a clear induction in LLC-challenged *Igf1r*^*fl/fl*^ mice, while both remained unaltered in *CreERT2* mice (Fig. 3B). The proportion of circulating neutrophils and monocytes exhibited a marked increase in LLC-challenged *Igf1r*^*fl/fl*^ mice, and while the proportion of neutrophils remained high, monocyte counts did not change in *CreERT2* mice after LLC challenge. In contrast, we observed a significant reduction in the proportion of lymphocytes in LLC-challenged mice, and this reduction was lower in *CreERT2* mice. Circulating levels of eosinophils did not change between experimental groups (Fig. 3C, D). On the other hand, a significant increase in total cell numbers and neutrophil counts was observed in bone marrow of *Igf1r*^*fl/fl*^ mice after LLC challenge, an increase that was not significant in LLC-challenged IGF1R-deficient mice (Fig. 3C, E). Moreover, total and differential BALF cell counts for neutrophils, macrophages and lymphocytes were found elevated in LLC-challenged *Igf1r*^*fl/fl*^ mice, while this increase was less pronounced in *CreERT2* mice. Total protein concentration in BALF was significantly increased in LLC-challenged *Igf1r*^*fl/fl*^ mice but remained unaltered in *CreERT2* mice upon LLC challenge (Fig. 3C, F).

As a complement to the LLC experimental pulmonary metastasis model, we generated an additional mouse model: B16-F10 (melanoma) cells were intravenously injected in IGF1R-deficient (*CreERT2*) and *Igf1r*^*fl/fl*^ mice to induce lung metastasis (Supplementary Fig. 1A). As noted in the LLC model, TNFα levels in both serum and lung homogenates also demonstrated a clear induction in B16-F10-challenged *Igf1r*^*fl/fl*^ mice, remaining unaltered in *CreERT2* mice (Supplementary Fig. 1B). Accordingly, total and differential BALF cell counts for macrophages and lymphocytes, as well as total protein concentration in BALF were found elevated in B16-F10-challenged *Igf1r*^*fl/fl*^ mice, but not in *CreERT2* mice as shown in the LLC model. Neutrophil counts in BALF did not show any significant changes between groups, unlike in the LLC model (Supplementary Fig. 1C, D).

### Reduced tumor burden and decreased expression of metastasis markers, p-IGF1R and p-ERK1/2, as well as changes in IGF system gene expression in lungs of IGF1R-deficient mice

**Fig. 4:**
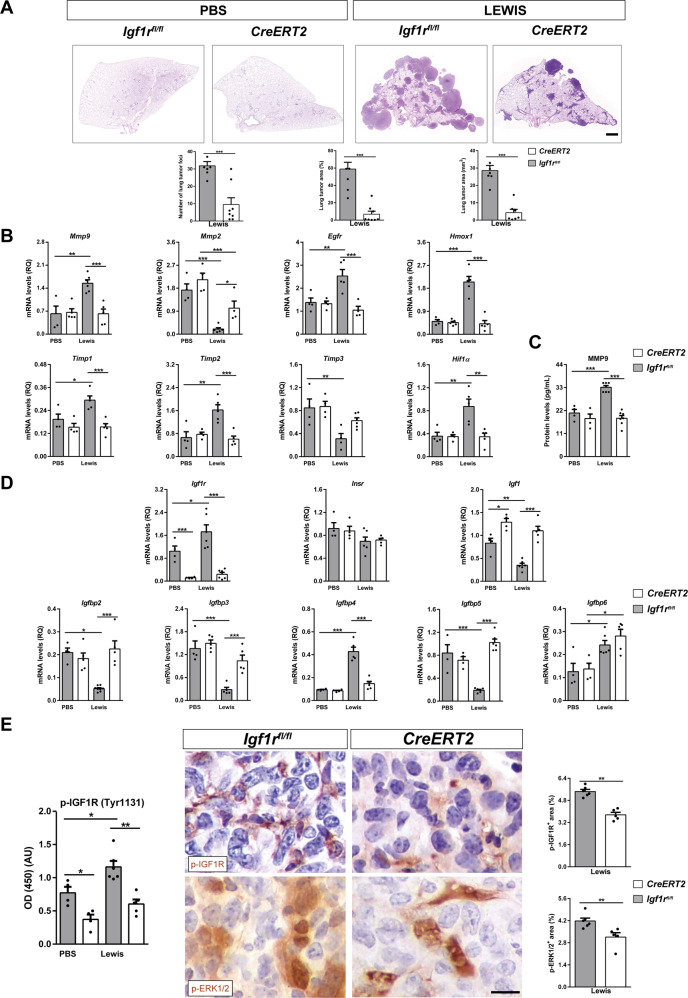
Reduced tumor burden and decreased expression of metastasis markers, p-IGF1R and p-ERK1/2, as well as changes in IGF system gene expression in lungs of IGF1R-deficient mice. **A** Representative histopathology images of lung metastasis (H&E) and respective quantifications of lung foci and lung tumor area (% and mm^2^) in IGF1R-deficient (*CreERT2*) vs. *Igf1r*^*fl/f*^ mice (*n* = 5–8 mice per group; Scale bar: 100 µm). **B** Lung tissue mRNA expression levels of *Mmp9*, *Mmp2*, *Egfr* and *Hmox1* (tumor progression), *Timp2* and *Timp3* (inhibitors of metalloproteinases), and *Hif1α* (hypoxia) markers, normalized to 18 S expression in PBS- or LLC-challenged, and **C** MMP9 protein levels in lung homogenates of *CreERT2* vs. *Igf1r*^*fl/fl*^ mice (*n* = 4–7 mice per group). **D** Lung tissue mRNA expression of IGF system-related genes *Igf1r, Insr, Igf1, Igfbp2, Igfbp3*, *Igfbp4, Igfbp5* and *Igfbp6* normalized to 18 S expression in PBS- or LLC-challenged *CreERT2* vs. *Igf1r*^*fl/f*^ mice (*n* = 5–7 mice per group). **E** p-IGF1R protein levels in lung homogenates, as well as representative immunostains for p-IGF1R and p-ERK1/2 (p-42/44) and respective quantifications of p-IGF1R^+^ and p-ERK1/2^+^ areas (%) (brown) in lung metastatic tumors of PBS- or LLC-challenged *CreERT2* vs. *Igf1r*^*fl/fl*^ mice (*n* = 4–6 mice per group; Scale bar: 15 µm). Quantifications were performed randomly in five different fields. Data are expressed as mean ± SEM. *p < 0.05; **p < 0.01; ***p < 0.001 (Mann-Whitney U test or Student´s t-test for comparing two groups and the Dunn-Sidak test for multiple comparisons).

To evaluate the effect of IGF1R depletion on LLC metastasis, tumor burden, metastasis markers, p-IGF1R, p-ERK1/2 and IGF system gene expression were assessed in lungs of IGF1R-deficient (*CreERT2*) mice vs. controls. After LLC experimental pulmonary metastasis, *CreERT2* mice exhibited decreased lung tumor foci and area with respect to *Igf1r*^*fl/fl*^ mice (Fig. 4A). A similar result was found in mice challenged with B16-F10 cells (Supplementary Fig. 1E). mRNA expression of *Mmp9, Egfr* and *Hmox1* (tumor progression), *Timp1* and *Timp2* (inhibitors of metalloproteinases) and *Hif1α* (hypoxia) evaluated in lung homogenates was significantly augmented in LLC-challenged *Igf1r*^*fl/fl*^ mice, remaining unaltered in *CreERT2* mice. Conversely, *Mmp2* (tumor progression) and *Timp3* (inhibitor of metalloproteinases) mRNA expression was significantly repressed after LLC challenge in *Igf1r*^*fl/fl*^ mice, while this repression was milder in *CreERT2* mice (Fig. 4B). Accordingly, MMP9 levels quantified by ELISA in lung homogenates mirrored its mRNA expression profile (Fig. 4C). Regarding IGF system gene expression, *Igf1r* mRNA expression increased significantly in *Igf1r*^*fl/fl*^ mice upon experimental pulmonary metastasis, showing an efficient depletion in PBS- and LLC-challenged *CreERT2* mice, as expected due to tamoxifen-mediated *Igr1r* gene depletion. Insulin receptor (*Insr)* mRNA levels did not change between experimental groups. In contrast, *Igf1* mRNA levels showed significantly increased levels in both *CreERT2* experimental groups indicating IGF1 resistance to the IGF1R-defiency condition. Surprisingly, a significant reduction in *Igf1* mRNA levels was noticed in *Igf1r*^*fl/fl*^ mice upon experimental pulmonary metastasis. Notably, mRNA expression of *Igfbp* genes *Igfbp2, Igfbp3* and *Igfbp5* was found significantly depleted, and *Igfbp4* levels significantly increased upon LLC challenge only in *Igf1r*^*fl/fl*^ mice. Specifically*, Igfbp6* expression was slightly increased within LLC experimental groups (Fig. 4D). p-IGF1R levels assessed by ELISA in lung homogenates showed a significant increase in *Igf1r*^*fl/fl*^ mice after experimental pulmonary metastasis, but were reduced in IGF1R-deficient PBS- and LLC-challenged mice. On the other hand, p-IGF1R and p-ERK1/2, assessed by immunohistochemistry in metastatic tumors, demonstrated smaller stained areas in LLC-challenged *CreERT2* mice (Fig. 4E).

### IGF1R deficiency decreases proliferation, DNA damage, senescence, and vascularization, attenuates tumor invasion by reduced EMT and fibrosis, and induces apoptosis upon pulmonary metastasis

**Fig. 5:**
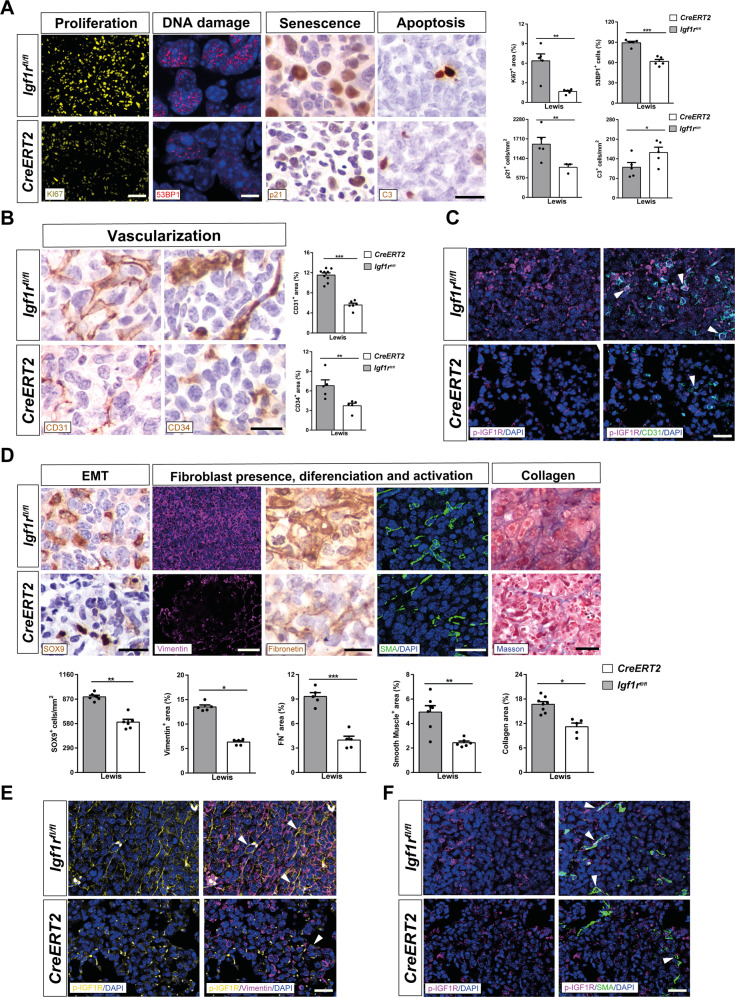
IGF1R deficiency decreases proliferation, DNA damage, senescence and vascularization, attenuates tumor invasion by reduced EMT and fibrosis, and induces apoptosis upon pulmonary metastasis. **A** Representative immunostains and quantifications of Ki67^+^ (proliferation) (yellow), 53BP1^+^ (DNA damage) (red) area (%), p21^+^ (senescence) (brown) and C3^+^ (apoptosis) (brown) cells per unit area (mm^2^), as well as **B** CD31^+^ (vascularization) and CD34^+^ (differentiated vascularization) (brown) areas (%) in the lung TME of LLC-challenged *CreERT2* vs. *Igf1r*^*fl/fl*^ mice (n = 5-7 mice per group; Scale bars: 30 µm in ki67, 8 µm in 53BP1, and 15 µm rest of immunostains). **C** Representative double immunostains for p-IGF1R and CD31 (magenta and green, respectively; white arrowheads indicate colocalization) in the lung TME of LLC-challenged *CreERT2* vs. *Igf1r*^*fl/fl*^ mice (*n* = 3–5 mice per group; Scale bars: 20 µm). **D** Representative immunostains for SOX9 (EMT) (brown), Vimentin (fibroblast presence) (magenta), Fibronectin (fibroblast differentiation) (brown), and SMA (fibroblast activation) (green), and stains for Masson (collagen content), as well as number of SOX9^+^ cells per unit area (mm^2^) and Vimentin^+^, Fibronectin^+^, SMA^+^ and Masson^+^ areas (%) in the lung TME of LLC-challenged *CreERT2* vs. *Igf1r*^*fl/f*^ mice (*n* = 5–6 mice per group; Scale bars: 15, 30, 15, 20 and 30 µm, respectively). **E, F** Representative double immunostains for p-IGF1R and Vimentin (yellow and magenta; white arrowheads indicate colocalization) and for p-IGF1R and SMA (magenta and green; white arrowheads indicate colocalization) in the lung TME of LLC-challenged *CreERT2* vs. *Igf1r*^*fl/fl*^ mice (*n* = 3–5 mice per group; Scale bars: 20 µm). Quantifications were performed randomly in five different fields. Data are expressed as mean ± SEM. *p < 0.05; **p < 0.01; ***p < 0.001 (Mann–Whitney U test or Student´s *t*-test for comparing two groups).

**Fig. 6:**
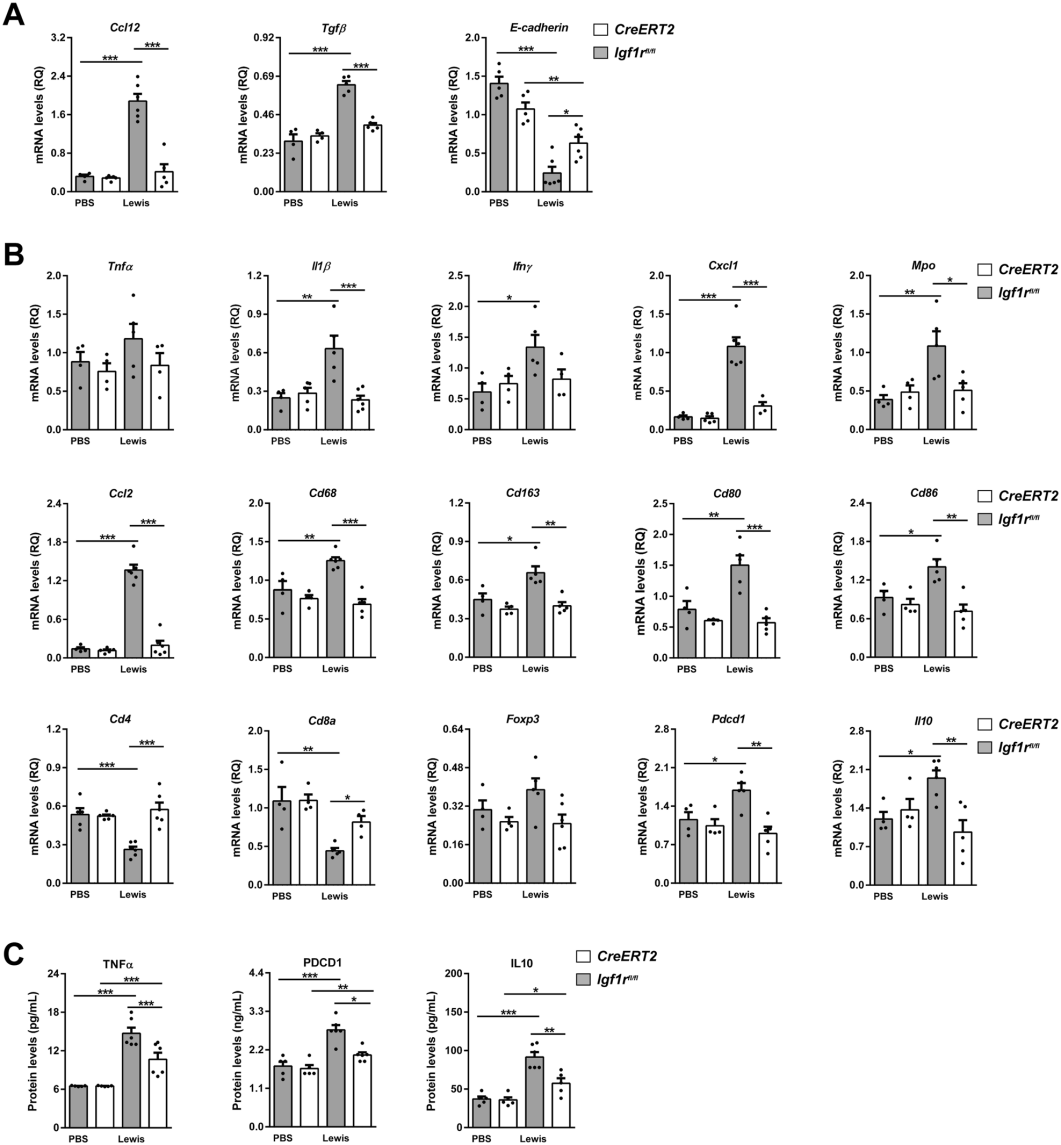
IGF1R depletion diminishes expression of inflammation and lung tumor immunosuppression markers. **A** Lung tissue mRNA expression of *Ccl12* (recruitment of fibrocytes), *Tgfβ* and *E-cadherin* (epithelial-mesenchymal transition, EMT) normalized to 18 S expression (*n* = 4–6 mice per group) in lung homogenates from PBS- or LLC-challenged *CreERT2* vs. *Igf1r*^*fl/fl*^ mice. **B** Lung tissue mRNA expression levels of *Tnfα* and *Il1β* (Th1 inflammation), *Ifnγ* (T cell exhaustion), *Cxcl1* (neutrophil chemotaxis), *Mpo* (neutrophils), *Ccl2* (macrophage chemotaxis), *Cd68* and *Cd163* (tumor-associated macrophages, TAMs), Cd80 and Cd86 (dendritic cell activation), *Cd4, Cd8a* and *Foxp3* (tumor-infiltrating lymphocytes, TILs), *Pdcd1* (PD-1) (immunosuppression) and *Il10* (immunosuppression), normalized to 18 S expression in PBS- or LLC-challenged *CreERT2* vs. *Igf1r*^*fl/f*^ mice (*n* = 4–6 mice per group). **C** TNFα, PDCD1 (PD-1) and IL10 protein levels in lung homogenates from PBS- or LLC-challenged *CreERT2*) vs. *Igf1r*^*fl/fl*^ mice (*n* = 5–7 mice per group). Data are expressed as mean ± SEM. *p < 0.05; **p < 0.01; ***p < 0.001 (Dunn–Sidak test for multiple comparisons).

To determine the effect of IGF1R deficiency in the metastatic TME, we immunostained lung tumors of LLC-challenged mice for the following markers: Ki67 (proliferation), 53BP1 (DNA damage), p21 (senescence), C3 (apoptosis), CD31 and CD34 (vascularization), SOX9 (epithelial-mesenchymal transition, EMT), as well as Vimentin, Fibronectin, SMA and Masson (fibrosis). All markers showed decreased expression in LLC-challenged IGF1R-deficient *CreERT2* mice, except for C3 whose expression was found to be significantly increased (Fig. 5A, B, D). In accordance, quantification of immunostains for Ki67, CD31, Vimentin and SMA were also found decreased in B16-F10-challenged *CreERT2* mice with respect to *Igf1r*^*fl/fl*^ (Supplementary Fig. 1F). Moreover, to determine how the lack of IGF1R in the lung TME is mechanistically affecting tumor growth and metastasis, we performed double fluorescence immunostainings of p-IGF1R with CD31 (vascularization), Vimentin (fibroblast presence) and SMA (fibroblast activation) in lung sections from LLC-challenged mice. In this regard, representative immunostainings indicated a reduction of p-IGF1R expression in blood vessels and fibroblasts (Fig. 5C, E, F). To complement these data, we also assessed mRNA expression of *Ccl12* (recruitment of fibrocytes), *Tgfβ* and *E-cadherin* (EMT) markers in lung homogenates. Concerning EMT and fibrosis, lung mRNA levels of *Ccl12* and *Tgfβ* were found significantly increased in LLC-challenged *Igf1r*^*fl/fl*^ mice, remaining unaltered in *CreERT2* mice. In contrast, *E-cadherin* mRNA expression was highly attenuated in *Igf1r*^*fl/fl*^ mice upon experimental pulmonary metastasis, while this reduction was milder in *CreERT2* mice (Fig. 6A).

### IGF1R depletion diminishes inflammation and attenuates lung tumor immunosuppression

To evaluate the impact of IGF1R deficiency on lung inflammation and immunosuppression, total mRNA expression and protein levels of related markers were assessed on lung homogenates by qPCR and ELISA, as well as by immunostaining (Fig. 6B, C). Interestingly, mRNA levels of *Il1β, Ifnγ, Cxcl1, Mpo, Ccl2, Cd68, Cd163, Cd80, Cd86, Pdcd1 and Il10* were strongly induced after experimental pulmonary metastasis in *Igf1r*^*fl/fl*^ mice, remaining unaltered in IGF1R-deficient mice (Fig. 6B). Exceptionally, *Tnfα* and *Foxp3* markers were found slightly augmented, and *Cd4* and *Cd8a* strongly reduced in *Igf1r*^*fl/fl*^ mice upon LLC challenge. Accordingly, TNFα, PDCD1 (PD-1) and IL10 protein levels were significantly increased in *Igf1r*^*fl/fl*^ mice upon LLC challenge, while this increase was milder in *CreERT2* mice (Fig. 6C).

**Fig. 7:**
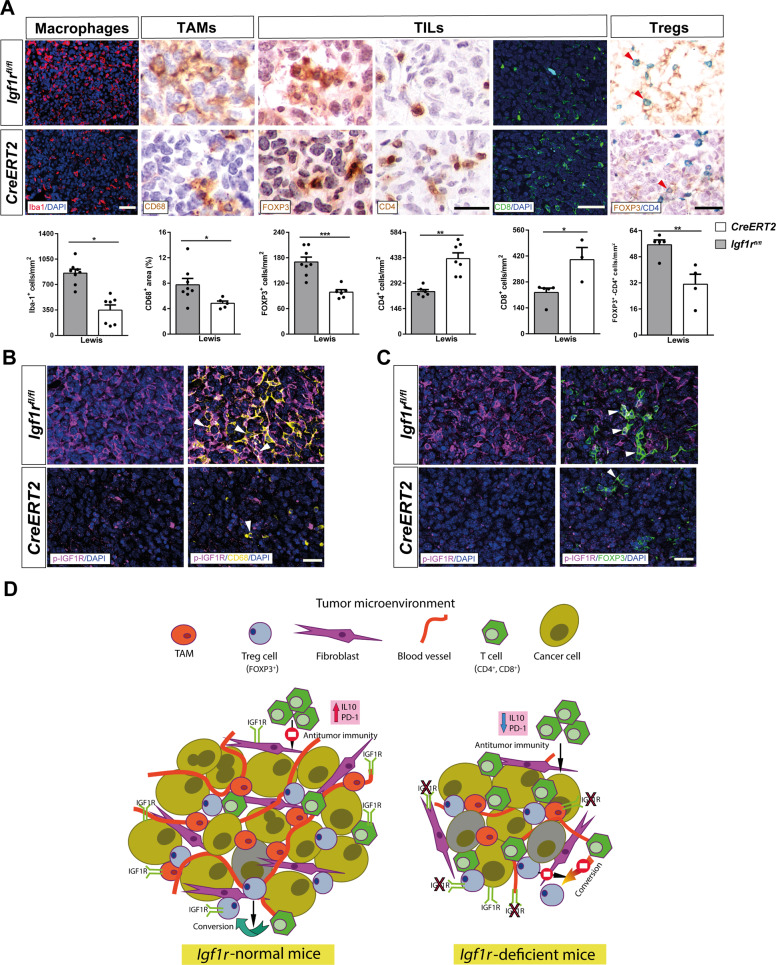
IGF1R deficiency reduces inflammation and attenuates lung tumor immunosuppression. **A** Representative immunostains and quantification of CD68^+^ (TAMs) (brown) area (%), Iba1^+^ (macrophages) (red), FOXP3^+^ (brown), CD4^+^ (brown), and CD8^+^ (green) (TILs), and FOXP3^+^CD4^+^ (Tregs) (green-magenta; red arrowheads indicate colocalization) cells per unit area (mm^2^) in the lung TME of LLC-challenged *CreERT2* vs. *Igf1r*^*fl/fl*^ mice (n = 3-6 mice per group; Scale bars: 30, 25, 40 and 25 µm, respectively). **B, C** Representative double immunostains for p-IGF1R and CD68 (magenta and yellow) and for p-IGF1R and FOXP3 (magenta and green; white arrowheads indicate colocalization) in the lung TME of LLC-challenged *CreERT2* vs. *Igf1r*^*fl/fl*^ mice (n = 4-5 mice per group; Scale bars: 30 µm). Quantifications were performed randomly in five different fields. Data are expressed as mean ± SEM. *p < 0.05; **p < 0.01; ***p < 0.001 (Mann–Whitney U test or Student´s *t*-test for comparing two groups). **D** *Proposed mechanism for reduced lung metastasis implantation and progression in IGF1R-deficient mice*. Following induction of lung metastasis, IGF1R deficient mice exhibited reduced tumor burden, increased apoptosis, diminished proliferation, vascularization and EMT and fibrosis, as well as attenuated inflammation and immunosuppression. In accordance, IGF1R deficiency decreased expression of p-IGF1R in blood vessels, fibroblasts, TAMs and FOXP3^+^ TILs, key components in the lung TME. Specifically, IGF1R deficiency decreased the presence of TAMs and FOXP3^+^ TILs, which are known to promote lung tumor progression, as well as reduced IL10 and PD-1 levels, thus stimulating antitumor immunity. Moreover, decreased infiltration of FOXP3^+^ Tregs in IGF1R-deficient lungs could also reduce the conversion of antitumor CD4^+^ T cells into FOXP3^+^ Tregs.

To reinforce these data, we also performed immunostainings for Iba1 (macrophages), CD68 (TAMs), FOXP3, CD4 and CD8 (TILs), as well as double immunostainings for FOXP3 and CD4 (Tregs) in lung sections from LLC-challenged mice. We observed a decreased presence of Iba1, CD68, FOXP3 and double FOXP3-CD4 positive cells, along with an increased number of CD4 and CD8 positive cells in LLC-challenged *CreERT2* mice (Fig. 7A). Similarly, we also consistently found a decreased presence of Iba1^+^ macrophages in B16-F10 metastatic tumors from *CreERT2* mice (Supplementary Fig. 1G). In addition, to determine how the lack of IGF1R in the lung TME is conditioning tumor growth and metastasis, we performed double fluorescence immunostainings of p-IGF1R with CD68 (TAMs) and FOXP3 (FOXP3^+^ TILs) in lung sections from LLC-challenged mice. In this regard, representative immunostainings indicated an apparent reduction of p-IGF1R expression in TAMs and FOXP3^+^ TILs (Fig. 7B, C). Altogether, our findings suggest that IGF1R depletion in the lung TME of IGF1R-deficient mice delays lung metastasis implantation and progression by reducing the presence of blood vessels, fibroblasts, TAMs and FOXP3^+^ TILs, as well as by increasing the amount of infiltrating CD4^+^ and CD8^+^ T cells (Fig. 7D).

## Discussion

We aimed to determine how IGF1R deficiency acts in the lung tumor microenvironment (TME) conditioning metastatic tumor implantation and progression, by generating LLC models using heterotopic transplantation or pulmonary metastasis in the context of IGF1R deficiency. We also explored genomic alterations of IGF1R in patients with NSCLC, as well as IGF1R protein expression and levels in tissue samples and serum from NSCLC patients.

We found increased serum IGF1R levels in our NSCLC patient cohort. IGF1R was previously found in serum and plasma exosomes [17, 18], and recently identified as a novel plasma biomarker to predict mortality in COVID-19 patients [19]. Thus, IGF-1R could be a candidate serum biomarker for the NSCLC diagnosis. We also report IGF1R overexpression in NSCLC patient lung tissues, in accordance with previous reports where its upregulation was associated with reduced disease-free survival [12, 16]. Accordingly, data obtained from cBioPortal cancer database showed increased gene amplification frequency, mRNA expression and copy number values of *IGF1R* in NSCLC patient tissue samples.

Since the Lewis lung carcinoma (LLC) model is the only reproducible syngeneic model for NSCLC [15], we deemed it is appropriate for determining the effect of IGF1R deficiency on key components of the TME, not only using an experimental pulmonary metastasis model but also upon heterotopic syngeneic transplantation. LLC primary tumors generated in IGF1R-deficient mice showed delayed tumor implantation and progression. Overall, these results indicate that IGF1R deficiency could have an antitumoral effect on the lung TME. After experimental pulmonary metastasis, IGF1R-deficient mice exhibited reduced tumor implantation in both LLC and melanoma models, as similarly observed upon heterotopic syngeneic transplantation*. CreERT2* mice also showed decreased total protein concentration in BALF, an indicator of reduced vascular permeability, as reported in an additional IGF1R-deficient mouse line [20], which supports diminished presence of inflammatory cells in the lung. In this regard, *CreERT2* mice were reported to confer resistance to initiation of the inflammatory response [21–23]. In addition, IGF1/IGF1R signaling in the TME was found to be critical for medulloblastoma growth [24]. Furthermore, peripheral IL6 and TNFα levels were found reduced in *CreERT2* mice upon induction of LLC metastasis. Thus, elevated serum IL6 and TNFα levels were found associated with tumor recurrence in NSCLC patients [25].

LLC-challenged IGF1R-deficient mice exhibited unchanged lung mRNA expression of *Mmp9*, *Egfr*, *Hmox1*, *Hif1-α*, *Timp1, Timp2* and *Timp3*. MMP-9 was reported to promote LLC cell invasiveness and pulmonary metastasis, and was found overexpressed NSCLC patients [26, 27]. Of relevance, EGFR increased in NSCLC patients, and MMP9 and EGFR co-expression was associated with a poor prognosis [28, 29]. Moreover, HMOX1/HO-1 (heme oxygenase 1) was reported to promote lung metastasis in mice, and its high expression was correlated with tumor invasiveness in NSCLC, as similarly reported for HIF-1α [30–32]. In addition, TIMP1 overexpression or TIMP3 silencing have been linked to cancer progression and poor prognosis in NSCLC [33]. The unexpected reduction of *Mmp2* mRNA expression observed in LLC-challenged *Igf1r*^*fl/fl*^ mice does not correlate with MMP2 overexpression reported in NSCLC patients [34]. It should be noted that differences in mRNA expression patterns between *Mmp9* and *Mmp2* upon LLC challenge observed in IGF1R-deficient mice could be related to their distinct functional implication in this context, since MMP9 but not MMP2 was reported to promote LLC tumorigenesis [26].

As expected, LLC-challenged IGF1R-deficient mice exhibited counteracted IGF1R phosphorylation (p-IGF1R) and diminished p-ERK1/2 levels. Accordingly, IGF1R has been reported to contribute to the pathogenesis of lung cancer, as it is commonly overexpressed in NSCLC patients [12, 16]. On the other hand, p-ERK1/2 is a major IGF1R MAP kinase signaling mediator that was extensively reported to be activated in NSCLC and associated with tumor cell proliferation [35, 36]. Intriguingly, we found unchanged lung mRNA expression of *Insr* in all experimental groups, as well as reduced *Igf1* expression in *Igf1r*^*fl/fl*^ LLC-challenged mice. Of note, *Insr* expression was found upregulated in mouse lungs with compromised IGF1R signaling [21–23, 37], and IGF1 expression by the TME was reported to have a supportive role in tumor initiation and progression [16, 24]. These discrepancies could be due to differential transcriptional and post-transcriptional regulation of *Insr* and *Igf1* between different mouse models or consequence of the complex interactions and regulation between the insulin and IGF system components. Noteworthy, *Igf1* expression was found increased in PBS and LLC-challenge IGF1R deficient mice with respect to *Igf1r*^*fl/fl*^ mice. These results indicate that IGF1R deficiency itself confers IGF1 resistance. Accordingly, we have previously reported a similar *Igf1* mRNA expression profile in IGF1R deficient mice [22], and Moody et al. have reported increased circulating levels of IGF1 after IGF1R blockade [38]. We think that discrepancies between increased *Igf1* expression and resistance to devolvement of LLC in IGF1R-deficient mice could also be due to differential transcriptional regulation of *Igf1*. Specifically, in pancreatic and breast cancer models, macrophages and fibroblasts were shown to be the main producers of IGF1, supporting drug resistance and metastasis of cancer cells [39, 40]. Here we show that IGF1R deficiency in mice reduced p-IGF1R expression in blood vessels, fibroblasts, TAMs and Tregs, supporting reduced metastasis observed in IGF1R deficient mice. Our results demonstrate that p-IGF1R expression is apparently reduced in the lung TME of IGF1R-deficient mice, suggestive of a functionally “weak” TME, contributing to a reduced lung metastasis implantation and progression. Accordingly, inactivation of IGF1R was recently reported to delay tumor progression [24]. Even though *Igfbp2*, *Igfbp3* and *Igfbp5* expression was reduced, and in the case of *Igfbp4* increased upon LLC challenge, IGF1R deficiency maintained its levels unchanged. Moreover, *Igfbp6* expression was found slightly incremented upon LLC challenge in both genotypes. Concerning IGFBPs, we have previously reported that IGFBP2-6 are highly expressed at the transcriptional level in lungs of young mice [37]. Specifically, we previously reported similar expression profiles showing transcriptional repression of *Igfbp3* and *Igfbp5* after different challenges in IGF1R-deficient lungs [21–23, 37]. It should be noted that due to its complex regulation at different levels, IGFBPs were described to promote or suppress tumor growth in various tissues and contexts [41]. Specifically, IGFBP3 was reported to inhibit tumorigenesis and cell growth, and IGFBP5 was suggested to function as a tumor suppressor [42–45]. Conversely, IGFBP4 overexpression was found adversely associated with the prognosis of lung cancer patients [46]. On the other hand, the role of both IGFBP2 and IGFBP6 in cancer remains less clearly understood [41].

Interestingly, after LLC-challenge, IGF1R deficient mice showed increased apoptosis. Accordingly, IGF1R signaling was reported to protect tumor cells from apoptosis [47]. Moreover, IGF1R deficiency also reduced proliferation, DNA damage, senescence, vascularization, EMT and fibrosis in the lung TME, most of which are considered cancer hallmarks [6]. Remarkably, increased DNA damage was found associated with NSCLC development. Assessment of undifferentiated (CD31^+^) and differentiated (CD34^+^) blood vessels are important prognostic factors in advanced NSCLC [48–50]. EMT, a key indicator of early stage NSCLC, was reported to promote tumor growth and invasion, and was associated with increased IGF1R expression [51]. EMT is manifested by the loss of E-cadherin and increased expression of Vimentin and SOX9, thus promoting tumor invasion [52, 53]. Accordingly, these markers were found counteracted in *CreERT2* mice after LCC challenge. Furthermore, IGF1R deficiency also attenuated TGFβ, an EMT promoter favoring tumor invasion, metastasis, and transdifferentiation of cancer-associated fibroblasts (CAFs). SMA, a classical marker of CAFs, was found reduced in *CreERT2* mice, as well as fibronectin that was shown to stimulate NSCLC growth [54–57].

As expected, LLC-challenged *Igf1r*^*fl/fl*^ lungs showed augmented levels of the *Il1β, Ifnγ, CD80, CD86*, TNFα and IL10 inflammation markers that were consistently counteracted in IGF1R-deficient mice. TNF-α, IL1B, IFN-γ and IL10 were reported to have immunoevasive and T cell exhaustion functions, and to promote tumorigenesis, drug resistance and poor survival in NSCLC [25, 58–60]. Moreover, CD80^+^ and CD86^+^ dendritic cells present in peritumoral tissues of NSCLC patients were associated with an immature phenotype that favors tumor immune escape [61]. Within the lung TME many cells modulate the antitumor response including CD68^+^ TAMs, FOXP3^+^ Tregs, CD4^+^ helper T cells and CD8^+^ cytotoxic T cells [62]. Here we demonstrate that IGF1R deficiency decreased the presence of macrophages, TAMs, Tregs and neutrophils, and their respective chemotactic chemokines, as well as increased the presence of CD4^+^ and CD8^+^ cells upon LLC induction. The TME of NSCLC contains a large number of TAMs which influence tumor progression and patient prognosis, and specifically, M2 TAMs were shown to induce tumor aggressiveness and proliferation in NSCLC [63, 64]. In this respect, ablation of IGF1R to reduce M2 marker expression in the murine myeloid lineage was reported [65]. Moreover, accumulation of M2 TAMs in solid tumors was associated with hypoxia (HIF1α), IL-10 and TGFβ production [66, 67], as well as cytokines that were found counteracted in the TME of IGF1R- deficient mice. Of note, FOXP3 was reported to promote tumor growth and metastasis by inducing EMT in NSCLC [68]. Moreover, accumulation of CD4^+^FOXP3^+^ Tregs was reported to correlate with increasing incidence of lung cancer [69]. Interestingly, expression of *Igf1r* was found upregulated in Treg cells and IGF1R-deficient Treg cells were reported to express lower levels of FOXP3 [70, 71]. On the other hand, the presence of infiltrating CD8^+^ and CD4^+^ T cells is a favorable prognostic factor in NSCLC, since their activation correlates with a stronger antitumor immune response [72, 73]. Specifically, we report counteracted TNFα levels in IGF1R-deficient mice upon LLC challenge. In this regard, blocking of TNFα was shown to enhance CD8 T-cell-dependent immunity, thus favoring accumulation of CD8^+^ TILs [74, 75]. In addition, CD4 and CD8 immunity in NSCLC patients was reported to be required for clinical responses to PD-1 [76, 77]. PD-1 is a key element within the TME and therefore an important mechanism of tumor-immune resistance, thus PD-1 blockade was proposed as an attractive therapy in NSCLC [62, 78]. Herein we show that LLC-challenged *CreERT2* mice exhibited reduced inflammation and attenuated tumor immunosuppression by depleted PD-1 levels. Accordingly, Ajona et al. have recently demonstrated that both genetic and pharmacological inhibition of the IGF-1/IGF-1R axis enhance the antitumor activity of anti-PD-1–PD-L1 antibodies against lung cancer [16]. Of note, this is the first report showing that IGF1R acts in the lung TME sustaining inflammation and tumor-associated immunosuppression.

In summary, our results demonstrate that IGF1R deficiency in the lung TME impairs tumor initiation and progression (proposed mechanism in Fig. 7D). Our research indicates that IGF1R could be a potential biomarker for early prediction of drug response and clinical evolution of NSCLC patients.

## Materials and methods

### Clinical samples

Genomic data on amplification frequency, mRNA expression and copy number values of *IGF1R* in tissue samples from NSCLC patients were obtained from the cBio Cancer Genomics Portal (cBioPortal) (http://www.cbioportal.org). Formalin-fixed paraffin-embedded lung cancer tissues from 14 NSCLC patients were obtained from the San Pedro Hospital (Logroño, Spain). Serum samples from 24 NSCLC patients and matched controls were obtained from the Fundación Jimenez Díaz Hospital (Madrid, Spain). All patients provided written informed consent for participation in the study. Serum IGF1R levels were measured using human IGF1R ELISA kit. For additional details, see supplementary information.

### Cell lines and culture conditions

LLC/1 (Lewis Lung Carcinoma) and B16-F10 (Melanoma) cell lines were cultured following the American Type Culture Collection (http://www.atcc.org) recommendations and standard methods. For additional details, see supplementary information.

### Mice and ethical statement

For experimental purposes, *UBC-Cre-ERT2;Igf1r*^*fl/fl*^ mice were crossed with *Igf1r*^*fl/fl*^ mice to directly generate descendants in equal proportions in the same litter. Tamoxifen (TMX) was administered daily for five consecutive days to four-week-old mice of both genotypes to induce a postnatal *Igf1r* gene conditional deletion [79].

All experiments and animal procedures were carried out in accordance with the European Communities Council Directive guidelines (86/609/EEC) and were revised and approved by the CEAA/CIBIR (Gobierno de La Rioja) Bioethics Committee (refs. JGP02_1, JGP02_7 and JGP02_9). For additional details, see supplementary information.

### Heterotopic syngeneic transplantation

Nine- to 10 week-old (W9-10) female mice of both genotypes (*Igf1r*^*fl/fl*^ and *UBC-Cre-ERT2; Igf1r*^*fl/fl*^ (*CreERT2*)) were subcutaneously injected in shaved flanks with 1 × 10^6^ Lewis Lung Carcinoma (LLC) cells in PBS (100 µl) or equal volume PBS at day (D) 0, under light isoflurane anesthesia. LLC engraftments were allowed to grow for 14 days (D14). Tumor size was measured with a caliper on alternate days. Tumor volumes were determined using the formula: volume = (width^2^ × length) × 0.50 [80], and their weight assessed at D14. Mice were euthanized by intraperitoneal injection of 10 μL/g of a ketamine-xylazine anesthetic combination in saline (300:30 mg/kg, respectively). Resected tumors were fixed by inflation with 4% formaldehyde for 8–10 h and embedded in paraffin for immunohistochemistry.

### Experimental pulmonary metastasis

Ten- to 12 week-old (W10-12) female mice of both genotypes (*Igf1r*^*fl/fl*^ and *UBC-Cre-ERT2;Igf1r*^*fl/fl*^ (*CreERT2*)) were injected through the lateral tail vein with 1 ×10^6^ LLC or B16-F10 cells in PBS (100 µL) or equal volume PBS at D0 under light isoflurane anesthesia.

### Sample collection and preparation

Animals were euthanized using 10 μL/g ketamine-xylazine. Blood was then collected, and lungs were lavaged with PBS. Right lung lobes were dissected and snap frozen for quantitative PCR (qPCR) and ELISA analyses, and left lung lobes were harvested for histopathological evaluation or immunohistochemistry. Femurs were dissected to isolate bone marrow. For additional details, see supplementary information.

### Quantification of blood, BALF and bone marrow cells

Total cell number was counted and expressed as cells/mL in BALF and BM, and as a percentage in peripheral blood. Cells were determined to be macrophages, lymphocytes and neutrophils using standard morphology criteria [81]. For additional details, see supplementary information.

### Histopathological and immunostaining analysis

Hematoxylin and eosin (H&E) staining was performed to quantify the number of surface metastases and to evaluate lung tumor area. Masson´s trichrome staining was for quantifying collagen deposition. IGF1R, CD45, p-IGF1R, p-ERK1/2 (p-42/44), Ki67, CD31, CD34, Vimentin, Fibronectin, SMA and CD68 antibodies were used to evaluate IGF1R^+^, CD45^+^, p-IGF1R^+^, p-ERK1/2^+^ (p-42/44), Ki67^+^, CD31^+^, CD34^+^, Vimentin^+^, Fibronectin^+^, SMA^+^ and CD68^+^ areas. 53BP1, C3, p21, SOX9, Iba1, FOXP3 and CD4 antibodies were used to determine the number of 53BP1^+^, C3^+^, p21^+^, SOX9^+^, Iba1^+^, FOXP3^+^ and CD4^+^ cells. For additional details, see supplementary information.

### RNA isolation, reverse transcription, and qPCR

Inferior right lung lobes were homogenized in TRIzol, and RNA was isolated and reverse-transcribed to cDNA. cDNA samples were amplified by qPCR for each primer pair assayed (Supplementary Table 2). Results were normalized using the 18 S rRNA gene (*Rn18s*). For additional details, see supplementary information.

### Mouse ELISAS

Total serum IL6 and TNFα levels were assessed with mouse ELISA kits. Superior right lung lobes were homogenized in RIPA Buffer. Phospho(p)-IGF1R, MMP9, IL10, TNFα and PD-1 levels were evaluated in homogenized lung tissue lysates using mouse ELISA kits, and normalized to total protein levels. *F*or additional details, see supplementary information.

### Statistics

Statistical analyses were carried out using SPSS Statistics Software v21 for Windows (IBM, Armonk, NY). Following a Shapiro-Wilk normality test, the statistical significance was determined using the Mann-Whitney U test or Student´s t-test for comparing two groups. According to the sample distribution, either a one-way ANOVA test or a Kruskal–Wallis test were used and then, the post hoc Dunn–Sidak test was carried out for multiple comparisons. For all analysis, a *p* value <0.05 was considered statistically significant.

## Supplementary information

Supplementary information

## Data Availability

Data available on request from the authors.

## References

[1] Siegel RL, Miller KD, Jemal A (2021). Cancer statistics. CA Cancer J Clin.

[2] American Cancer Society. Cancer Facts & Figures 2021. Atlanta: American Cancer Society; 2021. (https://www.cancer.org/research/cancer-facts-statistics/all-cancer-facts-figures/cancer-facts-figures-2021.html).

[3] Rangachari D, Brahmer JR (2013). Targeting the immune system in the treatment of non-small-cell lung cancer. Curr Treat Options Oncol.

[4] Gerull WD, Puri V, Kozower BD (2021). The epidemiology and biology of pulmonary metastases. J Thorac Dis.

[5] Jamil A, Kasi A Lung metastasis. StatPearls 2021; Bookshelf ID:NBK553111. 31971751

[6] Hanahan D, Weinberg RA (2011). Hallmarks of cancer: the next generation. Cell.

[7] Peinado H, Zhang H, Matei IR, Costa-Silva B, Hoshino A, Rodrigues G (2017). Pre-metastatic niches: organ-specific homes for metastases. Nat Rev Cancer.

[8] Maru Y (2015). The lung metastatic niches. J Mol Med (Berl).

[9] Girnita L, Worrall C, Takahashi SI, Seregard S, Girnita A (2014). Something old, something new and something borrowed: Emerging paradigm of insulin-like growth factor type 1 receptor (IGF-1R) signaling regulation. Cell Mol Life Sci.

[10] Wang Z, Li W, Guo Q, Wang Y, Ma L, Zhang X (2018). Insulin-like growth factor-1 signaling in lung development and inflammatory lung diseases. Biomed Res Int.

[11] Griffiths CD, Bilawchuk LM, McDonough JE, Jamieson KC, Elawar F, Cen Y (2020). IGF1R is an entry receptor for respiratory syncytial virus. Nature.

[12] Nurwidya F, Andarini S, Takahashi F, Syahruddin E, Takahashi K (2016). Implications of insulin-like growth factor 1 receptor activation in lung cancer. Malays J Med Sci.

[13] Iams WT, Lovly CM (2015). Molecular pathways: clinical applications and future direction of insulin-like growth factor-1 receptor pathway blockade. Clin Cancer Res.

[14] Osher E, Macaulay VM (2019). Therapeutic targeting of the IGF axis. Cells.

[15] Kellar A, Egan C, Morris D (2015). Preclinical murine models for lung cancer: clinical trial applications. Biomed Res Int.

[16] Ajona D, Ortiz-Espinosa S, Lozano T, Exposito F, Calvo A, Valencia K (2020). Short-term starvation reduces IGF-1 levels to sensitize lung tumors to PD-1 immune checkpoint blockade. Nat Cancer.

[17] Yamaguchi T, Izumi Y, Nakamura Y, Yamazaki T, Shiota M, Sano S (2015). Repeated remote ischemic conditioning attenuates left ventricular remodeling via exosome-mediated intercellular communication on chronic heart failure after myocardial infarction. Int J Cardiol.

[18] He M, Crow J, Roth M, Zeng Y, Godwin AK (2014). Integrated immunoisolation and protein analysis of circulating exosomes using microfluidic technology. Lab Chip.

[19] Fraser DD, Cepinskas G, Patteron EK, Slessarev M, Martin C, Daley M (2020). Novel outcome biomarkers identified with targeted proteomic analyses of plasma from critically ill coronavirus disease 2019 patients. Crit Care Explor.

[20] Ahamed K, Epaud R, Holzenberger M, Bonora M, Flejou J-F, Puard J (2005). Deficiency in type 1 insulin-like growth factor receptor in mice protects against oxygen-induced lung injury. Respir Res.

[21] Piñeiro-Hermida S, Alfaro-Arnedo E, Gregory JA, Torrens R, Ruíz-Martínez C, Adner M (2017). Characterization of the acute inflammatory profile and resolution of airway inflammation after Igf1r-gene targeting in a murine model of HDM-induced asthma. PLoS One.

[22] Piñeiro-Hermida S, López IP, Alfaro-Arnedo E, Torrens R, Iñiguez M, Alvarez-Erviti L (2017). IGF1R deficiency attenuates acute inflammatory response in a bleomycin-induced lung injury mouse model. Sci Rep.

[23] Piñeiro-Hermida S, Gregory JA, López IP, Torrens R, Ruíz-Martínez C, Adner M (2017). Attenuated airway hyperresponsiveness and mucus secretion in HDM-exposed Igf1r-deficient mice. Allergy.

[24] Yao M, Ventura PB, Jiang Y, Rodriguez FJ, Wang L, Perry JSA (2020). Astrocytic trans-differentiation completes a multicellular paracrine feedback loop required for medulloblastoma tumor growth. Cell.

[25] Shang G-S, Liu L, Quin Y-W (2017). IL-6 and TNF-α promote metastasis of lung cancer by inducing epithelial-mesenchymal transition. Oncol Lett.

[26] Chou CH, Teng C-M, Tzen K-Y, Chang Y-C, Chen J-H, Cheng JC-H (2012). MMP-9 from sublethally irradiated tumor promotes Lewis lung carcinoma cell invasiveness and pulmonary metastasis. Oncogene.

[27] El-Badrawy MK, Yousef AM, Shaalan D, Elsamanoudy AZ (2014). Matrix metalloproteinase-9 expression in lung cancer patients and its relation to serum mmp-9 activity, pathologic type, and prognosis. J Bronchol Inter Pulmonol.

[28] Yang C-H, Chou H-C, Fu Y-N, Yeh C-L, Cheng H-W, Chang I-C (2015). EGFR over-expression in non-small cell lung cancers harboring EGFR mutations is associated with marked down-regulation of CD82. Biochim Biophys Acta.

[29] Cox G, Jones JL, O´Byrne KJ (2000). Matrix metalloproteinase 9 and the epidermal growth factor fignal pathway in operable non-small cell lung cancer. Clin Cancer Res.

[30] Lin H-H, Chiang M-T, Chang P-C, Chau L-Y (2015). Myeloid heme oxygenase-1 promotes metastatic tumor colonization in mice. Cancer Sci.

[31] Tsai J-R, Wang H-M, Liu P-L, Chen Y-H, Yang M-C, Chou S-H (2012). High expression of heme oxygenase-1 is associated with tumor invasiveness and poor clinical outcome in non-small cell lung cancer patients. Cell Oncol(Dordr).

[32] Yohena T, Yoshino I, Takenaka T, Kameyama T, Ohba T, Kuniyoshi Y (2009). Upregulation of hypoxia-inducible factor-1alpha mRNA and its clinical significance in non-small cell lung cancer. J Thorac Oncol.

[33] Jackson HW, Defamie V, Waterhouse P, Khokha R (2017). TIMPs: versatile extracellular regulators in cancer. Nat Rev Cancer.

[34] Passllick B, Sienel W, Seen-Hibler R, Wöckel W, Thetter O, Mutschler W (2000). Overexpression of matrix metalloproteinase 2 predicts unfavorable outcome in early-stage non-small cell lung cancer. Clin Cancer Res.

[35] Vicent S, López-Picazo JM, Toledo G, Lozano MD, Torre W, Garcia-Corchón C (2004). ERK1/2 is activated in non-small-cell lung cancer and associated with advanced tumours. Br J Cancer.

[36] López-Malpartida AV, Ludeña MD, Varela G, Pichel JG (2009). Differential ErbB receptor expression and intracellular signaling activity in lung adenocarcinomas and squamous cell carcinomas. Lung Cancer.

[37] López IP, Piñeiro-Hermida S, Pais RS, Torrens R, Hoeflich A, Pichel JG (2016). Involvement of Igf1r in bronchiolar epithelial regeneration: Role during repair kinetics after selective club cell ablation. PLoS One.

[38] Moody G, Beltran PJ, Mitchell P, Cajulis E, Chung Y-A, Hwang D (2014). IGF1R blockade with ganitumab results in systemic effects on the GH–IGF axis in mice. J Endocrinol.

[39] Ireland L, Santos A, Ahmed MS, Rainer C, Nielsen SR, Quaranta V (2016). Chemoresistance in pancreatic cancer is driven by stroma-derived insulin-like growth factors. Cancer Res.

[40] Ireland L, Santos A, Campbell F, Figueiredo C, Hammond D, Ellies LG (2018). Blockade of insulin-like growth factors increases efficacy of paclitaxel in metastatic breast cancer. Oncogene.

[41] Baxter RC (2014). IGF binding proteins in cancer: mechanistic and clinical insights. Nat Rev Cancer.

[42] Wang YA, Sun Y, Palmer J, Solomides C, Huang L-C, Shyr Y (2017). IGFBP3 modulates lung tumorigenesis and cell growth through IGF1 signaling. Mol Cancer Res.

[43] Alami N, Page V, Yu Q, Jerome L, Paterson J, Shiry L (2008). Recombinant human insulin-like growth factor-binding protein 3 inhibits tumor growth and targets the Akt pathway in lung and colon cancer models. Growth Horm IGF Res.

[44] Wang J, Ding N, Li Y, Cheng H, Wang D, Yang Q (2015). Insulin-like growth factor binding protein 5 (IGFBP5) functions as a tumor suppressor in human melanoma cells. Oncotarget.

[45] Le HT, Lee HJ, Cho J, Min H-Y, Lee J-S, Lee S-J (2021). Insulin-like growth factor binding protein-3 exerts its anti-Metastatic effect in aerodigestive tract cancers by disrupting the protein stability of vimentin. Cancers (Basel).

[46] Xiao Y, Zhu S, Yin W, Liu X, Hu Y (2017). IGFBP‑4 expression is adversely associated with lung cancer prognosis. Oncol Lett.

[47] Yuan J, Yin Z, Kaixiong T, Wang G, Gao J (2018). Function of insulin-like growth factor 1 receptor in cancer resistance to chemotherapy. Oncol Lett.

[48] Orlow I, Park BJ, Mujumdar U, Patel H, Siu-Lau P, Clas BA (2008). DNA damage and repair capacity in patients with lung cancer: prediction of multiple primary tumors. J Clin Oncol.

[49] Zhao Y-Y, Xue C, Jiang W, Zhao H-Y, Huang Y, Feenstra K (2012). Predictive value of intratumoral microvascular density in patients with advanced non-small cell lung cancer receiving chemotherapy plus bevacizumab. J Thorac Oncol.

[50] Bačić I, Karlo R, Zadro AS, Zadro Z, Skitarelić N, Antabak A (2018). Tumor angiogenesis as an important prognostic factor in advanced non-small cell lung cancer (Stage IIIA). Oncol Lett.

[51] Chen B, Xiao F, Li B, Xie B, Zhou J, Zheng J (2013). The role of epithelial-mesenchymal transition and IGF-1R expression in prediction of gefitinib activity as the second-line treatment for advanced nonsmall-cell lung cancer. Cancer Invest.

[52] Yang Y-L, Chen M-W, Xian L (2014). Prognostic and clinicopathological significance of downregulated E-cadherin expression in patients with non-small cell lung cancer (NSCLC): a metaanalysis. PLoS One.

[53] Huang J-Q, Wei F-K, Xu X-L, Ye S-X, Song J-W, Ding P-K (2019). SOX9 drives the epithelial-mesenchymal transition in non-small-cell lung cancer through the Wnt/β-catenin pathway. J Transl Med.

[54] Kim BN, Ahn DH, Kang N, Yeo CD, Kim YK, Lee KY (2020). TGF‑β induced EMT and stemness characteristics are associated with epigenetic regulation in lung cancer. Sci Rep..

[55] Calon A, Tauriello DVF, Batle E (2014). TGF-beta in CAF-mediated tumor growth and metastasis. Semin Cancer Biol.

[56] Schulze AB, Schulze AB, Schmidt LH, Heitkötter B, Huss S, Mohr M (2020). Prognostic impact of CD34 and SMA in cancer-associated fibroblasts in stage I-III NSCLC. Thorac Cancer.

[57] Han SW, Khuri FR, Roman J (2006). Fibronectin stimulates non-small cell lung carcinoma cell growth through activation of Akt/mammalian target of rapamycin/S6 kinase and inactivation of LKB1/AMP-activated protein kinase signal pathways. Cancer Res.

[58] Mojic M, Takeda K, Hayakawa Y (2017). The dark side of IFN-ɤ: Its role in promoting cancer immunoevasion. Int J Mol Sci.

[59] Zaidi MR (2019). The interferon-gamma paradox in cancer. J Interferon Cytokine Res.

[60] Sung W-W, Wang Y-C, Lin P-L, Cheng Y-W, Chen C-Y, Wu T-C (2013). IL-10 promotes tumor aggressiveness via upregulation of CIP2A transcription in lung adenocarcinoma. Clin Cancer Res.

[61] Perrot I, Blanchard D, Freymond N, Isaac S, Guibert B, Pachéco Y (2007). Dendritic cells infiltrating human non-small cell lung cancer are blocked at immature stage. J Immunol.

[62] Nevee SC, Robinson BW, Fear VS (2019). The role and therapeutic implications of T cells in cancer of the lung. Clin Transl Immunol.

[63] Sumimoto R, Hirai T, Fujita M, Murakami H, Otake Y, Huang C-L (2019). M2 tumor-associated macrophages promote tumor progression in non-small-cell lung cancer. Exp Ther Med.

[64] Li Z, Maeda D, Yoshida M, Umakoshi M, Nanjo H, Shiraishi K (2018). The intratumoral distribution influences the prognostic impact of CD68- and CD204-positive macrophages in non-small cell lung cancer. Lung Cancer.

[65] Spadaro O, Camell CD, Bosurgi L, Nguyen KY, Youm Y-H, Rothlin CV (2017). IGF1 shapes macrophage activation in response to immunometabolic challenge. Cell Rep..

[66] Chen Y, Song Y, Du W, Gong L, Chang H, Zou Z (2019). Tumor-associated macrophages: an accomplice in solid tumor progression. J Biomed Sci.

[67] Zheng X, Weigert A, Reu S, Guenther S, Mansouri S (2020). Spatial density and distribution of tumor-associated macrophages predict survival in non-small-cell lung carcinoma. Cancer Res.

[68] Yang S, Liu Y, Li M-Y, Ng CSH, Yang S-L, Wang S (2017). FOXP3 promotes tumor growth and metastasis by activating Wnt/β-catenin signaling pathway and EMT in non-small cell lung cancer. Mol Cancer.

[69] Hou P-F, Zhu L-J, Chen X-Y, Qiu Z-Q (2017). Age-related changes in CD4+CD25+FOXP3+ regulatory T cells and their relationship with lung cancer. PLoS One.

[70] DiToro D, Harbour SN, Bando KJ, Benavides G, Witte S, Laufer VA (2020). Insulin-like growth factors are key regulators of T helper 17 regulatory T cell balance in autoimmunity. Inmunity.

[71] Bilbao D, Luciani L, Johannesson B, Piszczek A, Rosenthal N (2014). Insulin-like growth factor-1 stimulates regulatory T cells and suppresses autoimmune disease. EMBO Mol Med.

[72] Hiraoka K, Miyamoto M, Cho Y, Suzuoki M, Oshikiri T, Nakakubo Y (2006). Concurrent infiltration by CD8þ T cells and CD4þ T cells is a favourable prognostic factor in non-small-cell lung carcinoma. Br J Cancer.

[73] Goc J, Germain C, Vo-Bourgais TKD, Lupo A, Klein C, Knockaert S (2014). Dendritic cells in tumor-associated tertiary lymphoid structures signal a Th1 cytotoxic immune contexture and license the positive prognostic value of infiltrating CD8+ T cells. Cancer Res.

[74] Bertrand F, Rochotte J, Colarcios C, Montfort A, Tilkin-Mariamé A-F, Touriol C (2015). Blocking tumor necrosis factor α enhances CD8 T-cell-dependent immunity in experimental melanoma. Cancer Res.

[75] Bertrand F, Montfort A, Marcheteau E, Imbert C, Gilhodes J, Filleron T (2017). TNFα blockade overcomes resistance to anti-PD-1 in experimental melanoma. Nat Commun.

[76] Zuazo M, Arasanz H, Fernández-Hinojal G, García-Granda MJ, Gato M, Bocanegra A (2019). Functional systemic CD4 immunity is required for clinical responses to PD-L1/PD-1 blockade therapy. EMBO Mol Med.

[77] Tumeh PC, Harview CL, Yearley JF, Shintaku PI, Taylor EJM, Robert L (2014). PD-1 blockade induces responses by inhibiting adaptive immune resistance. Nature.

[78] Brahmer J, Reckamp KL, Baas P, Crinò L, Eberhardt WEE, Poddubskaya E (2015). Nivolumab versus docetaxel in advanced squamous-cell non-small-cell lung cancer. N. Engl J Med.

[79] López IP, Rodriguez-de la Rosa L, Pais RS, Piñeiro-Hermida S, Torrens R, Contreras J (2015). Differential organ phenotypes after postnatal Igf1r gene conditional deletion induced by tamoxifen in UBC-CreERT2; Igf1r fl/fl double transgenic mice. Transgenic Res.

[80] O´Reilly MS, Boehm T, Shing Y, Fukai N, Vasios G, Lane WS (1997). Endostatin: an endogenous inhibitor of angiogenesis and tumor growth. Cell.

[81] Alfaro-Arnedo E, López IP, Piñeiro-Hermida S, Ucero AC, González-Barcala FJ, Salgado FJ (2021). IGF1R as a potential pharmacological target in Allergic Asthma. Biomedicines.

