# Supplementary material for: IGF1R acts as a cancer-promoting factor in the tumor microenvironment facilitating lung metastasis implantation and progression
Source: Oncogene. 2022 Jun 10;41(28):3625–39. doi: 10.1038/s41388-022-02376-w (PMC9184253; doi:10.1038/s41388-022-02376-w)
Supplement: Supplementary file 1 — Supplementary information [file 41388_2022_2376_MOESM1_ESM.docx]

**Supplementary information for**

**IGF1R acts as a cancer-promoting factor in the tumor microenvironment facilitating lung metastasis implantation and progression**

**Elvira Alfaro-Arnedo,^1^ Icíar P. López,^1,#^ Sergio Piñeiro-Hermida,^2,#^ Marta Canalejo, ^1^ Carolina Gotera,^3,4^ Jesús Javier Sola,^5^ Alejandra Roncero,^6^ Germán Peces-Barba,^3,4^ Carlos Ruíz-Martínez,^6^ José G. Pichel,^1,4*^**

^1^Lung Cancer and Respiratory Diseases Unit, Center for Biomedical Research of La Rioja (CIBIR), Fundación Rioja Salud, Logroño, Spain.

^2^Telomeres and Telomerase Group, Molecular Oncology Program, Spanish National Cancer Centre (CNIO) Madrid, Spain.

^3^IIS Fundación Jiménez Díaz, Madrid, Spain

^4^Spanish Biomedical Research. Networking Centre-CIBERES, Madrid

^5^Pathological Anatomy Service, Hospital Universitario San Pedro, Rioja Salud, Logroño, Spain.

^6^Pneumology Service, Hospital Universitario San Pedro, Rioja Salud, Logroño, Spain.

^#^These authors contributed equally to this work

**Files included:**

**Supplementary Fig. 1** IGF1R deficiency reduces the number of lung tumor foci, leukocytes in BALF, proliferation, vascularization, fibrosis and macrophage presence in the TME, and attenuates the increase of TNFα levels upon lung melanoma metastasis.

**Supplementary Table 1** Increased amplification frequency of *IGF1R* in NSCLC patients.

**Supplementary Table 2** Primer sets used for qPCR.

**Supplementary Methods**

**Supplementary Fig. 1 IGF1R deficiency reduces the number of lung tumor foci, leukocytes in BALF, proliferation, vascularization, fibrosis and macrophage presence in the TME, and attenuates the increase of TNFα levels upon lung melanoma metastasis.** (**A**) *UBC-CreERT2;Igf1r^fl/fl^* and *Igf1r^fl/fl^* female mice were treated with tamoxifen (TMX) for five consecutive days at four weeks (W) of age to induce *Igf1r* gene deletion (*CreERT2*). Then, mice were injected through the lateral tail vein with 1 x 10^6^ B16-F10 (melanoma) cells in PBS or equal volume of PBS. Collection of BALF and lungs were performed on day (D) 14. (**B**) TNFα levels in both serum and lung homogenates from PBS- or B16-F10-challenged *CreERT2* vs. *Igf1r^fl/fl^* mice (n = 5-6 mice per group). (**C**-**D**) Total cells, neutrophils, macrophages and lymphocytes, as well as, total protein content (**D**) in BALF from PBS- or B16-F10-challenged *CreERT2* vs. *Igf1r^fl/fl^* mice (n = 5-7 mice per group). (**E**) Representative histopathology images of lung metastasis (H&E) and respective quantifications of number of lung foci and tumor area (% and mm^2^) in IGF1R-deficient mice (*CreERT2*) vs. *Igf1r^fl/fl^* mice (n = 5-7 mice per group; Scale bar: 100 µm). (**F**-**G**) Representative immunostains and quantification of Ki67^+^ (proliferation) (brown), CD31^+^ (vascularization) (brown), Vimentin^+^ (fibroblast presence) (magenta) and SMA^+^ (fibroblast activation) (green) areas (%), as well as Iba1^+^ (macrophages) (red) cells per unit area (mm^2^) (**G**), in the lung TME of B16-F10-challenged *CreERT2* vs. *Igf1r^fl/fl^* mice (n = 5-7 mice per group; Scale bars: 50 µm). Quantifications were performed in five different fields in a random way. Data are expressed as mean ± SEM. *p<0.05; **p<0.01; ***p<0.001 (Mann-Whitney U test or Student´s t-test for comparing 2 groups and the Dunn-Sidak test for multiple comparisons).

**Supplementary Table 1** Increased amplification frequency of *IGF1R* in NSCLC patients.

| **Reference/source** | **Pathology** | **No. of patients** | **Amplification frequency (%)** |
| --- | --- | --- | --- |
| (1) Campbell et al. Nat Genet 2016, 48(6):607-616 | Pan-lung cancer | 1144 | 17 (1.49 %) |
| (2) The Cancer Genome Atlas, Firehose Legacy | Lung squamous cell carcimoma | 502 | 17 (3.39 %) |
| (3) The Cancer Genome Atlas, PanCancer Atlas | Lung squamous cell carcimoma | 487 | 12 (2.46 %) |
| (4) The Cancer Genome Atlas, PanCancer Atlas | Lung adenocarcinoma | 566 | 5 (0.88 %) |
| (5) Jordan et al. Can Discov 2017, 7(6):596-609 | Non-small cell lung cancer | 915 | 2 (0.22 %) |
| (6) The Cancer Genome Atlas, Firehose Legacy | Lung adenocarcinoma | 516 | 7 (1.36 %) |
| (7) Memorial Sloan Kettering Cancer Center, MSK-IMPACT 2020 | Lung adenocarcinoma | 604 | 1 (0.17 %) |
| (8) Cancer Genome Atlas Research Network, Nature 2014, 511(7511):543-550 | Lung adenocarcinoma | 230 | 3 (1.3 %) |
| (9) Cancer Genome Atlas Research Network, Nature 2012, 489(7417):519-525 | Lung squamous cell carcimoma | 178 | 4 (2.25 %) |
| (10) Chen et al. Nat Genet 2020, 52(2):177-186 | Lung adenocarcinoma | 302 | 1 (0.33 %) |

**Supplementary Table 2** Primer sets used for qPCR.

| **Gene** | **Accession No.** | **Forward primer (5´-3´)** | **Reverse primer (5´-3´)** |
| --- | --- | --- | --- |
| ***Ccl2*** | NM_011333.3 | CACCAGCCAACTCTCACTGA | CGTTAACTGCATCTGGCTGA |
| ***Ccl12*** | NM_011331.3 | TCCTCAGGTATTGGCTGGAC | GGCTGCTTGTGATTCTCCTG |
| ***Cd163*** | NM_001170395.1 | TCTCCAGTCCAAACAACAAGC | ACCACCTCCACCTACCAAGC |
| ***Cd4*** | NM_013488.2 | ATGTGGAAGGCAGAGAAGGA | TGGGGTATCTTGAGGGTGAG |
| ***Cd8a*** | NM_001081110.2 | GGAGTGGAGAAGCTAAGCCA | TGGAGCTGGAGTTCTGGAAG |
| ***Cd68*** | NM_001291058.1 | TGTTCACCTTGACCTGCTCT | TTGCAAGAGAAACATGGCCC |
| ***Cd80*** | NM_001359898.1 | TATTGCTGCCTTGCCGTTAC | ACTCGGGCCACACTTTTAGT |
| ***Cd86*** | NM_019388.3 | GAAAGAGGAGCAAGCAGACG | TCTCCACGGAAACAGCATCT |
| ***Cxcl1*** | NM_008176.3 | ATCCAGAGCTTGAAGGTGTTG | GTCTGTCTTCTTTCTCCGTTACTT |
| ***E-cadherin*** | NM_009864.3 | CCAGCAGTTCGTTGTTGTCA | TGTGGAAGGGACAAGAGACC |
| ***Egfr*** | NM_207655.2 | ACAACCCCACCACCTATCAG | GCCATCTTCTTCCACTTCGT |
| ***Foxp3*** | NM_001199347.1 | CACCCAGGAAAGACAGCAAC | CTGCACCACTTCTCTCTGGA |
| ***Hif1α*** | NM_010431.2 | TTGGAACTGGTGGAAAAACTG | ACTTGGAGGGCTTGGAGAAT |
| ***Hmox1*** | NM_010442.2 | CACGCATATACCCGCTACCT | CCAGAGTGTTCATTCGAGCA |
| ***Ifnγ*** | NM_008337.4 | TTCTTCAGCAACAGCAAGGC | ACTCCTTTTCCGCTTCCTGA |
| ***Igf1*** | NM_010512 | cagaagcgatggggaaaat | gtgaaggtgagcaagcagag |
| ***Igf1r*** | NM_010513 | ATGGCTTCGTTATCCACGAC | AATGGCGGATCTTCACGTAG |
| ***Igfbp2*** | NM_008342 | GGGAGTGCTGGTGTGTGA | CTGCTGGTGTTCGGGATG |
| ***Igfbp3*** | NM_008343.2 | gccctctgccttcttgattt | tcactcggttatgggtttcc |
| ***Igfbp4*** | NM_010517.3 | TGTGAGATTGGATTGTGTGTGT | TAGAGATGGCGGGATAGGAG |
| ***Igfbp5*** | NM_010518.2 | GATGAGACAGGAATCCGAACAAG | AATCCT TTGCGGTCACAGTTG |
| ***Igfbp6*** | NM_008344 | AGGAGAGCAAACCCCAAGGA | TGAACAGGATTGGGCCGTATA |
| ***Il1β*** | NM_008361.3 | GCAACTGTTCCTGAACTCAACT | ATCTTTTGGGGTCCGTCAACT |
| ***Il10*** | NM_010548.2 | GCCTTATCGGAAATGATCCA | TTTTCACAGGGGAGAAATCG |
| ***Insr*** | NM_010568.2 | TCCTGAAGGAGCTGGAGGAGT | CTTTCGGGATGGCCTGG |
| ***Mmp2*** | NM_008610.3 | GATGTCGCCCCTAAAACAGA | GGTCTCGATGGTGTTCTGGT |
| ***Mmp9*** | NM_013599.4 | CCTGAAAACCTCCAACCTCA | GCTTCTCTCCCATCATCTGG |
| ***Mpo*** | NM_010824.2 | TGGTTGCCTGCAGAGTATGA | TCCTTGGTCAGCTGATCGTT |
| ***Pdcd1*** | NM_008798.2 | TCAAGGCATGGTCATTGGTA | GCTCCTCCTTCAGAGTGTCG |
| ***Rn18s*** | NR_003278.3 | ATGCTCTTAGCTGAGTGTCCCG | ATTCCTAGCTGCGGTATCCAGG |
| ***Timp1*** | NM_011593 | ATCTGGCATCCTCTTGTTGC | CTCGTTGATTTCTGGGGAAC |
| ***Timp2*** | NM_011594.3 | GCATCACCCAGAAGAAGAGC | GTCCATCCAGAGGCACTCAT |
| ***Timp3*** | NM_011595.2 | TAGAAGAGCAGGGCAGGAAG | GTCAGCACAGGGGAAAGATG |
| ***Tgfβ*** | NM_011577.2 | CGCAACAACGCCATCTATGA | ACTGCTTCCCGAATGTCTGA |
| ***Tnfα*** | NM_013693.3 | GCCTCTTCTCATTCCTGCTTG | CTGATGAGAGGGAGGCCATT |

**Supplementary Methods**

***Clinical samples***

Genomic data on amplification frequency, mRNA expression and copy number values of *Igf1r* in tissue samples from NSCLC patients were obtained from the The cBio Cancer Genomics Portal (cBioPortal) (http:// [www.cbioportal.org](http://www.cbioportal.org)), an online platform which provides visualization, analysis and download of large-scale cancer genomics data sets [1]. mRNA expression was assessed using an Illumina HiSeq sequencing system, as indicated in the cBioPortal website. Copy number variation (CNV) data were obtained as previously described [2]. Genomic libraries were obtained upon extraction, purification and digestion of genomic DNA from tissue samples of NSCLC patients. Such DNA was further hybridized onto Affymetrix SNP6.0 arrays and normalized as previously described [2]. Recurrent peaks for focal somatic copy number alteration were identified using GISTIC 2.0. A peak was considered focally amplified within a tumor if the GISTIC 2.0-estimated focal copy number ratio was greater than 0.1 [2]. Formalin-fixed paraffin-embedded lung cancer tissues from 14 NSCLC patients were obtained from the Hospital San Pedro (Logroño, Spain). Serum samples from 24 NSCLC patients and matched controls were obtained from the Hospital Fundación Jimenez Díaz (Madrid, Spain). All patients gave written informed consent. Study protocols were approved by the Ethics Committee of Clinical Research of La Rioja (CEICLAR, ref. PI-205), and by the Fundación Jiménez Díaz (CEImJGD, ref. ER_EO180-19_FJD-HGV). Serum IGF1R levels were measured using human IGF1R (Elabscience, Houston, TX) ELISA kit.

***Cell lines and culture conditions***

LLC/1 (Lewis Lung Carcinoma) and B16-F10 (Melanoma) cell lines were cultured following the American Type Culture Collection (http://www.atcc.org) recommendations and standard methods. These cell lines were maintained in DMEM + L-Glutamine (Gibco; Thermo Fisher Scientific, Inc., Waltham, MA) or RPMI 1640 (Lonza; Basel, Switzerland) media, respectively, supplemented with 10% fetal bovine serum (Gibco; Thermo Fisher Scientific) and penicillin (100 U mL^−1^) and streptomycin (100 μg mL^−1^). The cultures were maintained under an humidified atmosphere of 95% air / 5% CO2 at 37 °C and subcultured before they became confluent using a 0.25% trypsin/EDTA solution.

***Mice and ethical statement***

*UBC-Cre-ERT2;Igf1r^fl/fl^* double transgenic mouse line was in a C57BL/6 enriched (at least six generation backcrosses to C57BL/6 strain) mixed genetic background. For experimental purposes, *UBC-Cre-ERT2;Igf1r^fl/fl^* mice were crossed with *Igf1r^fl/fl^* mice to directly generate descendants in equal proportions in the same litter, and *Igf1r^fl/fl^* and *UBC-Cre-ERT2;Igf1r^fl/fl^* littermates were respectively used as experimental controls and mutants. Tamoxifen (TMX) was administered daily for five consecutive days to four-week-old mice of both genotypes to induce a postnatal *Igf1r* gene conditional deletion in *UBC-Cre-ERT2;Igf1r^fl/fl^* mice (*Cre-ERT2*) as previously described [3].

All experiments and animal procedures conducted were carried out in accordance with the guidelines of the European Communities Council Directive (86/609/EEC) and were revised and approved by the CEAA/CIBIR (Gobierno de La Rioja) Bioethics Committee (refs. JGP02_1, JGP02_7 and JGP02_9). All animals were bred and maintained under specific pathogen-free conditions at CIBIR animal facilities. The human endpoint criteria were applied when there was severe involvement of one of the specific (body weight reduction, stressed respiratory pattern or bleeding), or moderate involvement of two or more of the general (physical appearance or natural behaviour) or specific parameters occurred simultaneously.

***Sample Collection and Preparation***

Mice were euthanized by intraperitoneal injection of 10 μL/g of a ketamine-xylazine anesthetic combination in saline (300:30 mg/kg, respectively). Blood was collected by cardiac puncture, and then 50 µL were mixed with 1 mL of ACK Lysing Buffer (Thermo Fisher Scientific, Waltham, MA) and centrifuged at 300 xg for 5 min at 4°C after 15 min of incubation. Following aspiration of the supernatants, pellets were washed with 500 μL PBS and centrifuged at 300 xg for 5 min at 4°C, repeating this step once more. The supernatants were discarded and 200 μL of PBS were added to the pellets to prepare the cytospin preparations by centrifugation of the slides at 1500 rpm for 5 min (Cytospin 4, Thermo Fisher Scientific, Waltham, MA). Serum was obtained by centrifugation at 3000 xg for 10 min at 4°C and stored at -80°C until further use. Next, lungs were lavaged twice with 0.8 mL of cold PBS to obtain the bronchoalveolar lavage fluid (BALF) which was centrifuged at 15700 xg for 5 min at 4°C. The BALF supernatants were stored at -80°C to subsequently assess total protein concentration in BALF using the Pierce BCA Protein Assay Kit (Thermo Fisher Scientific). In addition, the BALF pellets were suspended in 500 μL ACK Lysing Buffer (Thermo Fisher Scientific) and centrifuged at 3300 xg for 5 min at 4°C after 10 min of incubation. The supernatants were discarded and 500 µL PBS were added to the pellet to prepare the cytospin preparations as mentioned above. Following lung dissection, right lung lobes were separated, snap-frozen in liquid nitrogen and stored at -80°C for quantitative PCR (qPCR) and ELISA analyses, and the left lung lobe was fixed by inflation with 4% formaldehyde for 8-10h, and subsequently embedded in paraffin for histopathology and immunohistochemistry. Bone marrow (BM) isolation was carried out following dissection of the femur. After centrifugation at 10000 xg for 15 seconds, BM was suspended in 500 μL PBS and centrifuged at 300 xg for 5 min at 4°C. Following aspiration of the supernatants, BM pellets were resuspended in 500 μL ACK Lysing Buffer (Thermo Fisher Scientific) and centrifuged at 300 xg for 5 min at 4°C after 10 min of incubation. The supernatants were discarded and 1 mL PBS was added to the pellets to prepare the cytospin preparations as mentioned above.

***Quantification of Blood, BALF and Bone Marrow Cells***

Total cell number was counted and expressed as cells/mL in BALF and BM, and as a percentage in peripheral blood. Differential cell counts were performed on May-Grünwald/Giemsa (Sigma-Aldrich, St. Louis, MO) stained cytospins, counting a minimum of 300 cells per slide in BALF, BM and blood cytospins.

***Histopathological and Immunostaining Analysis***

Paraffin-embedded engraftments and left lungs were cut into 3 μm sections for histology and immunohistochemistry. Hematoxylin and eosin (H&E) staining was performed to quantify the number of surface metastases and to evaluate lung tumor area (darker H&E stained foci). Masson´s trichrome staining was for quantifying collagen deposition (collagen area). Immunostaining was performed using the following antibodies: IGF1R (1:400, Boster Biological Technology Ltd., USA), 53BP1 (1:500, Novus Biologicals, Centennial, CO), Ki67 (Clone SP6 1:250, Master Diagnostica, Spain), C3 (Clone 5A1E 1:200, Abcam, Cambridge, UK), CD45 (Clone D3F8Q 1:900, Cell Signaling Technology, Danvers, MA), CD31 (1:50, Abcam), p-IGF1R (Clone Y1161 1:70, Abcam), p-ERK1/2 (p-42/44) (Clone E10 1:110, Cell Signalling Technology), p21 (Clone M19 1:110, Santa Cruz Biotech. Inc., Dallas, TX), CD34 (Clone RAM34 1:200, Thermo Fisher Scientific, Waltham, MA), Vimentin (Clone LN-6 1:400, Cell Signaling Technology), Fibronectin (1:100, Dako, Jena, Germany), Smooth Muscle Actin (SMA) (Clone 1A4 1:400, Sigma-Aldrich, St. Louis, MO), SOX9 (Clone E9 1:110, Santa Cruz Biotech. Inc.), Iba1 (1:800, Wako, Osaka, Japan), CD68 (Clone KP1 1:150, Santa Cruz Biotech. Inc.), FOXP3 (Clone 2A11G9 1:150, Santa Cruz Biotech. Inc.), CD4 (Clone D7D2Z 1:80, Cell Signaling Technology) and CD8 (Clone OX8 1:100, Santa Cruz Biotech. Inc.). IGF1R, p-IGF1R, p-ERK1/2 (p-42/44), Ki67, CD31, CD34, Vimentin, Fibronectin, SMA and CD68 antibodies were used to evaluate tumor IGF1R^+^, p-IGF1R^+^, p-ERK1/2^+^, Ki67^+^ (proliferation), CD31^+^ (vacularization), CD34^+^ (differentiated vacularization), Vimentin^+^ (fibroblast presence), Fibronectin^+^ (fibroblast diferentiation), SMA^+^ (fibroblast activation), and CD68^+^ (tumor associated macrophages) positive areas. 53BP1, p21, C3, SOX9, Iba1, FOXP3, CD4 and CD8 antibodies were used to determine the number of 53BP1^+^ (DNA damage), p21^+^ (senescence), C3^+^ (apoptosis), SOX9^+^ (epithelial mesenchymal transition), Iba1^+^ (macrophages), FOXP3^+^ (tumor infiltrating lymphocytes), CD4^+^ and CD8^+^ (T-cell markers) cells per unit area. Fiji opensource image processing software package v1.48r (http://fiji.sc) was used to quantify collagen deposition, IGF1R, p-IGF1R, p-ERK1/2, CD31, CD34, Fibronectin and CD68 positive areas (percentage of DAB), and Ki67, Vimentin and SMA positive areas (percentage of fluorescence). Quantifications in lung sections were performed in five different bronchi per animal in a random way.

***RNA Isolation, Reverse Transcription and qPCR***

Inferior right lung lobes were homogenized in TRIzol (Invitrogen, Carlsbad, CA), and RNA was isolated using an RNeasy Mini Kit (Qiagen, Hilden, Germany) and reverse-transcribed to cDNA using SuperScript II First-Strand Synthesis System (Invitrogen). cDNA samples were amplified by qPCR for each primer pair assayed (Table S2) in triplicate reactions for each primer pair assayed on a 7300 Real-Time PCR Instrument (Applied Biosystems, Foster City, CA), using SYBR Premix Ex Taq (Takara Bio Inc., Kusatsu, Japan). Results were normalized using the 18S rRNA gene (*Rn18s*).

***Mouse ELISAS***

Total serum IL6 and TNFα levels in mouse were assessed with the IL6 and TNFα Quantikine (R&D systems) ELISA kits. Superior right lung lobes were homogenized in RIPA Buffer (Thermo Scientific) containing a protease-phosphatase inhibitor mixture (Roche, Basel, Switzerland), and total protein concentration was determined with the Pierce BCA Protein Assay Kit (Thermo Fisher Scientific). Phospho(p)-IGF1R, MMP9, IL10, TNFα and PD-1 cytokine levels were determined in mouse lung lysates. p-IGF1R levels were quantified with the PathScanphospho-IGF-I receptor β (Tyr1131) sandwich ELISA kit (Cell Signaling Technology), and cytokines using mouse MMP9, IL10, TNFα and PD-1 Quantikine ELISA Kits (R&D systems, Minneapolis, MN) in volumes of lysates normalized to total lung protein levels.

**References**

1. Gao J, Aksoy BA, Dogrusoz U, Dresdner G, Gross B, Sumer SO, et al. Integrative analysis of complex cancer genomics and clinical profiles using the cBioPortal. Sci Signal. 2013;6(269):pl1.

2. Campbell JD, Alexandrov A, Kim J, Wala J, Berger AH, Pedamallu CS, et al. Distinct patterns of somatic genome alterations in lung adenocarcinomas and squamous cell carcinomas. Nat Genet. 2016;48(6):607-16.

3. López IP, Rodríguez-de la Rosa L, Pais RS, Piñeiro-Hermida S, Torrens R, Contreras J, et al. Differential organ phenotypes after postnatal Igf1r gene conditional deletion induced by tamoxifen in UBC-CreERT2; Igf1r fl/fl double transgenic mice. Transgenic Res. 2015;24:279-94.
